# Synthesis of High Entropy and Entropy-Stabilized Metal Sulfides and Their Evaluation as Hydrogen Evolution Electrocatalysts

**DOI:** 10.1021/acs.chemmater.3c00363

**Published:** 2023-09-19

**Authors:** Weichen Xiao, Yi Li, Amr Elgendy, Ercin C. Duran, Mark A. Buckingham, Ben F. Spencer, Bing Han, Firoz Alam, Xiangli Zhong, Sarah H. Cartmell, Robert J. Cernik, Alexander S. Eggeman, Robert A. W. Dryfe, David J. Lewis

**Affiliations:** † Department of Materials, The University of Manchester, Manchester M13 9PL, U.K.; ‡ Department of Chemistry, The University of Manchester, Oxford Road, Manchester M13 9PL, U.K.; § Egyptian Petroleum Research Institute, 11727 Cairo, Egypt

## Abstract

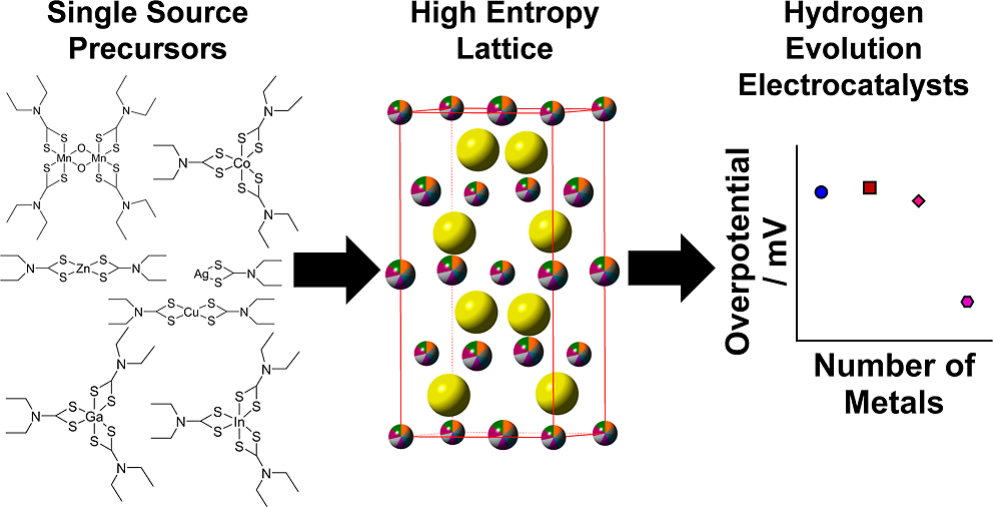

High entropy metal chalcogenides are materials containing five or more elements within a disordered sublattice. These materials exploit a high configurational entropy to stabilize their crystal structure and have recently become an area of significant interest for renewable energy applications such as electrocatalysis and thermoelectrics. Herein, we report the synthesis of bulk particulate HE zinc sulfide analogues containing four, five, and seven metals. This was achieved using a molecular precursor cocktail approach with both transition and main group metal dithiocarbamate complexes which are decomposed simultaneously in a rapid (1 h) and low-temperature (500 °C) thermolysis reaction to yield high entropy and entropy-stabilized metal sulfides. The resulting materials were characterized by powder XRD, SEM, and TEM, alongside EDX spectroscopy at both the micro- and nano-scales. The entropy-stabilized (CuAgZnCoMnInGa)S material was demonstrated to be an excellent electrocatalyst for the hydrogen evolution reaction when combined with conducting carbon black, achieving a low onset overpotential of (∼80 mV) and η_10_ of (∼255 mV).

## Introduction

Metal chalcogenides have been widely investigated for a range of applications such as electronics,^1,2^ photovoltaics,^3^ thermoelectrics,^4,5^ and photoelectrocatalysis.^6,7^ It can be surmised from this that metal chalcogenides are key material enablers for renewable energy technologies to reduce the current global reliance on fossil fuels and limit greenhouse gas emissions and climate change.^8^ Binary metal chalcogenides such as CdS and CdTe have been historically investigated for photoelectrochemical applications^6,9,10^ and Bi_2_Te_3_ for thermoelectric power conversion.^5,11^ However, research into multimetal chalcogenides has realized the synergistic benefit of several metals in ternary or quaternary systems such as CuZnSnS_4_ for photovoltaic and photoelectrochemical^12,13^ applications and AgSbTe_2_ for thermoelectric energy generation.^14,15^

Recently, this idea has been pushed ever further, inspired by the high-entropy nature of multimetal alloys,^16^ oxides, and halogens, which have all demonstrated enhanced stability and potency for catalysis.^17^ High entropy metal chalcogenides^18^ are a recent development that have already shown great promise in thermoelectric^19,20^ and electrocatalytic^21–23^ applications. This new class of metal chalcogenide is stabilized by a high configurational entropy of mixing of the constituent elements.^18^ Indeed, when a single crystalline phase can be formed from disparate elements that introduce disorder into the crystal lattice, these constitute special cases known as *entropy-stabilized systems*, where the entropic term becomes the dominating stabilizing force as opposed to traditional materials which are stabilized by lattice enthalpy. To date, there is still no universally accepted definition of when a material becomes a high entropy material, yet a single diffraction pattern is expected for entropy-stabilized systems, reflecting the stabilization of a single polymorph by entropy. Some attempts have been made to define high entropy materials, for example, it has been proposed that metal alloys become high entropy alloys when the material achieves a configurational entropy (*S*_conf_) > 1.5*R* (where *R* is the gas constant).^24^ Another report in high entropy alloys has suggested that any material with five or more principle elements can be classed as “high-entropy”.^25^ These materials are characterized as crystalline solids with an even distribution of constituent elements throughout the entire material. The high-entropy (HE) nature of these materials gives them advantageous properties such as frustrated thermal conductivity,^19^ large dielectric constants,^26^ and super ionic conductivity,^27^ toward applications such as thermoelectrics and electrocatalysis.

To date, there has only been a limited number of reports into the successful synthesis of high entropy/entropy-stabilized metal sulfides.^18^ These synthetic routes have required complicated, hazardous, time-consuming, and high temperature techniques. For example, one report requires over 60 h of ball milling^28^ to achieve a homogeneous distribution of the desired elements within the (CuSnMgGeZn)S and (CuSnMgInZn)S systems. Another method requires quartz annealing with severely hazardous HF for the reaction vessel preparation, followed by high temperature annealing of elemental powders over 120 h.^22^ Synthesis of (CrMnFeCoNi)S_*x*_ by pulsed thermal decomposition of metal salts and thiourea using an initial 6 h drying step, followed by very high annealing temperatures (∼1650 K) over short time scales (∼55 ms) and a rapid quenching mechanism has also been reported.^23^ To the best of our knowledge, only Schaak et al.^29^ have reported a low temperature synthetic method for high entropy Cu–Zn–Co–In–Ga–S. This was achieved through a cation exchange reaction of nanoparticulate Cu–S, which required several annealing steps, combined with Ar vacuum cycling. However, the scope of this type of synthesis is relatively narrow as it relies on the short diffusion lengths of the ions into nanoparticles and thus can probably not be applied to the synthesis of materials with dimensions >50 nm. For example, previous studies have determined the diffusion coefficients of metal ions in solids to be *ca*. 1 × 10^–9^ cm^2^ s^–1^ for Zn in Al at *ca*. 400 °C,^30^ or for Fe^2+^ in single crystal MgO at *ca*. 1650 °C.^31^ Using this value as a model for 1D diffusion of ions in solids suggests these ions will travel 15 nm in 25 min, consistent with the observations of Schaak et al.,^29^ whose prototype synthesis takes *ca*. 80 min. However, at this rate of diffusion, it would take ions *ca*. 115 days to travel 100 μm, which is not conducive to a synthetic procedure for bulk HE material. Therefore, to date, a simple, fast, direct, and low temperature synthetic route toward bulk HE metal sulfides remains elusive.

In order to address this gap in the synthetic toolkit toward these materials, here we report the tailored and scalable synthesis of bulk particulate high entropy metal sulfides. This was achieved using a molecular precursor cocktail approach with a low-temperature (500 °C) and fast (1 h) annealing step to decompose, in tandem, several homogeneously dispersed precursors to produce high entropy metal sulfides. The versatility of this approach has been demonstrated by synthesizing materials containing four, five, and seven metals; the latter is an example of a tailor-made entropy-stabilized system produced from a materials chemistry route. These particulate materials were characterized by powder XRD, SEM, and STEM analysis. Elemental mapping of the particles was measured on the microscale by SEM–EDX and on the nanoscale by STEM–EDX. The final configurational entropy of the synthesized materials was determined through quantitative SEM–EDX analysis. The entropy-stabilized (CuAgZnCoMnInGa)S system was used as an electrocatalyst for the hydrogen evolution reaction (HER). Low onset overpotential expressed as η_10_ (the overpotential where a current density of 10 mA cm^–2^ was obtained) were found in our entropy-stabilized system, compared to comparably synthesized quaternary systems [(CuInGa)S, (ZnInGa)S, and (CoInGa)S]. Attempts to deconvolute the activity of each material using a combination of electrochemical impedance spectroscopy (EIS) and electrochemically active surface area were undertaken and demonstrated that the excellent electrocatalytic performance of (CuAgZnCoMnInGa)S was not due to conventional effects such as high surface area. We therefore demonstrate that the unique properties resultant from the high entropy nature of the material is the cause of the high catalytic performance. In summary, a versatile, facile, fast, and low temperature approach toward bulk high entropy and entropy-stabilized metal sulfides is outlined.

## Experimental Section

### Synthesis

Synthesis of single source precursors was undertaken using metathesis of metal salts with sodium diethyldithiocarbamate. All syntheses were performed in atmospheric conditions; no special handling or inert conditions was required and are outlined in detail in the Supporting Information.

### Synthesis of High Entropy Sulfides Entropy-Stabilized Systems

Seven high entropy sulfides (HES) were successfully synthesized using the precursor decomposition approach. All metal dithiocarbamate precursors were combined in 10 mL of dichloromethane (DCM) to form a homogeneous solution, and the solvent was allowed to evaporate at room temperature and further removed under vacuum for 12 h prior to thermal decomposition. The final, homogeneously dispersed powder was then heated under an Ar atmosphere at 500 °C for 1 h.

The effect of altering decomposition time was undertaken on the (AgCuInGa)S system, where a decomposition at 450 °C was undertaken for both 1 and 5 h, under an Ar atmosphere.

The effect of altering decomposition temperature was also investigated on the (CuZnInGa)S system, where a 1 h decomposition was undertaken at 400, 450, and 500 °C under an Ar atmosphere. The five metal (CuZnCoInGa)S system produced at temperatures of 450, 500, and 550 °C for 1 h, under an Ar atmosphere were also examined.

The specific, synthesized HES can be subdivided into three groups:

#### Three-Metal Sulfides

The investigated metals for the three-metal systems were Cu, Co, Zn, In, and Ga. These were mixed in a 1:1:1: ratio of each respective precursor. Where 0.1 mmol of all metals was used as single source dithiocarbamate precursors. The produced ME materials were (CuInGa)S, (CoInGa)S, and (ZnInGa)S. (Cu_2_InGa)S was synthesized with 2 equiv Cu to 1 equiv In(DTC)_3_ and Ga(DTC)_3_.

#### Four-Metal Sulfides

The investigated metals for the four-metal systems were Cu, Ag, Zn, In, and Ga. These were mixed in a 1 (Cu) or 0.25 (Ag):1 (Zn):1 (In):1 (Ga) ratio. Where 0.1 mmol of all metals were used except Ag, which was 0.025 mmol—all as single-source dithiocarbamate precursors. The produced HE materials were (AgCuInGa)S, (CuZnInGa)S, and (AgZnInGa)S.

#### Five-Metal Sulfides

The investigated metals for five-metal systems were Cu, Ag, Mn, Co, Zn, In, and Ga. These were mixed in a 1 (Cu) or 0.25 (Ag):1 (Zn):1 (Mn):1 (In):1 (Ga) ratio. Where 0.1 mmol of all metals were used except Ag which was 0.025 mmol—all as single-source dithiocarbamate precursors. The produced HE materials were (CuZnMnInGa)S, (CuAgZnInGa)S, and (CuZnCoInGa)S.

#### Seven-Metal Sulfide

The seven-metal HES were investigated as 1 (Cu):0.5 (Ag):1 (Zn):1 (Co):1 (Mn):1 (In):1 (Ga), where 1 in these ratios represents 0.1 mmol metal dithiocarbamate precursor. The resultant product of this decomposition was expected to be (CuAgZnCoMnInGa)S.

### Characterization

The crystal structure of synthesized HES was examined by powder X-ray diffraction (*p*-XRD) using a Panalytical X’Pert Pro MPD diffractometer with Cu Kα radiation (λ = 0.15418 nm). The morphology and elemental distribution of the HES was investigated by FEI Quanta 650 SEM operating at 20 kV and 200 keV FEI Talos F200A for TEM. Thermogravimetric analysis (TGA) was conducted under N_2_ atmosphere from room temperature to 600 °C at a heating rate of 15 °C min^–1^ using a TGA STAR equipment (MettlerToledo). Detailed experimental results for both X-ray photoelectron spectroscopy (XPS) and electrochemical analysis are described in the Supporting Information.

## Results and Discussion

### Synthesis and p-XRD of High Entropy Metal Sulfides

**Figure 1:**
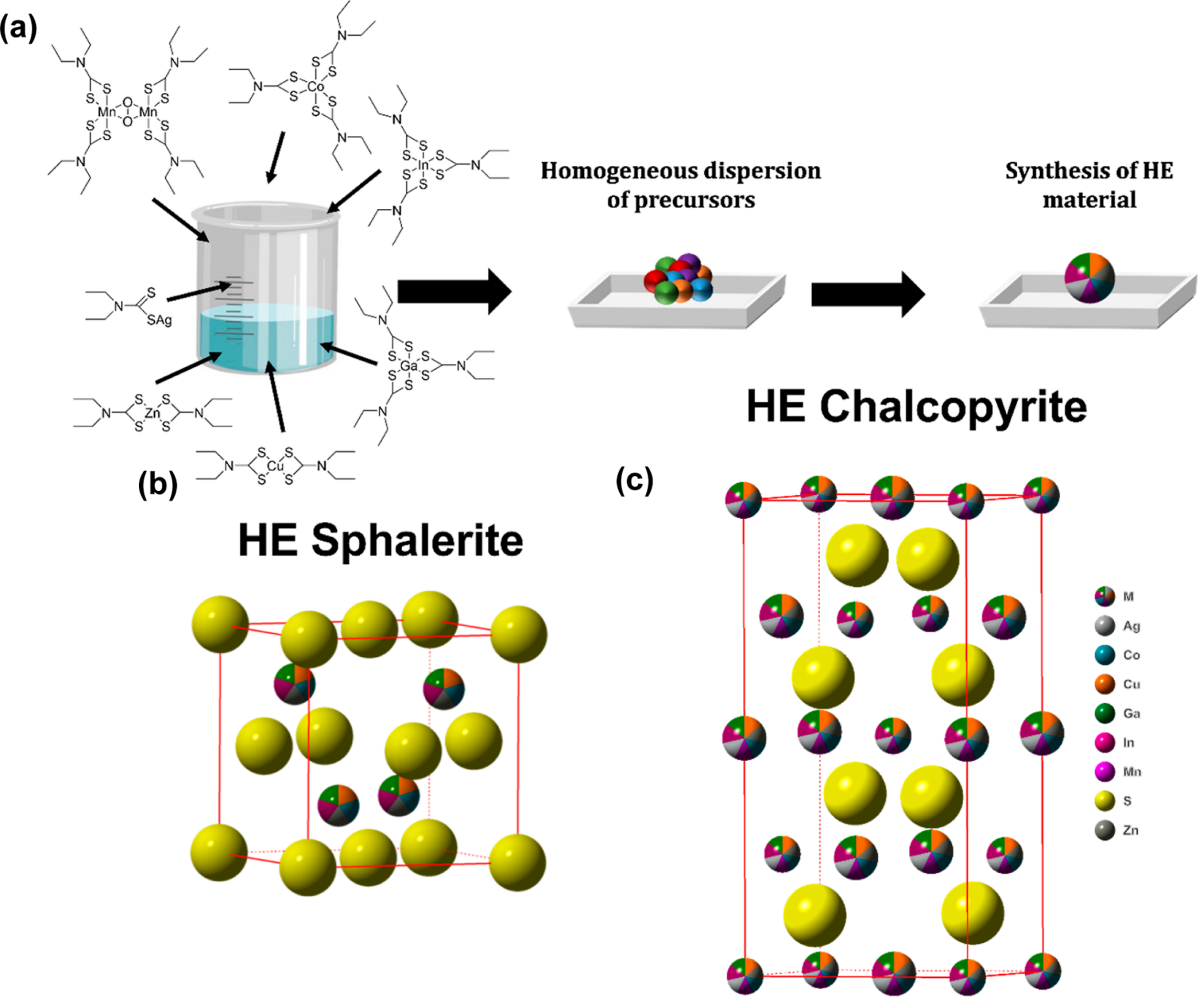
(a) schematic of the synthetic procedure of the HE metal sulfides. Initially all precursors are dissolved together in DCM which is subsequently evaporated to leave a homogeneous dispersion of precursors, this is followed by thermolysis in an inert atmosphere to yield the HE metal sulfides. (b) Unit cell of sphalerite (ICSD: 34529, *a* = 1.0940 Å, *b* = 6.3950 Å, *c* = 7.0220 Å, α = 90.000°, β = 121.173°, and γ = 90.000°), which the four- and five-metal sulfides all form. (c) Unit cell of chalcopyrite (ICSD: 2518, *a* = 5.2890 Å, *b* = 5.2890 Å, *c* = 10.4230 Å, α = 90.000°, β = 90.000°, γ = 90.000°) which the seven-metal sulfide forms. Metal positions within unit cells are shown as multicolored spheres and the sulfide positions as solid yellow spheres.

We set out to synthesize high entropy metal sulfides using various combinations of transition (Ag, Cu, Co, Mn, and Zn) and main group (In and Ga) metals. These high-entropy multimetal sulfides were synthesized through decomposition of metal diethyldithiocarbamates (structures of which are shown as insets in Figure S3).^32^ A schematic of this process is shown in Figure 1a, where the various combinations of metal sulfide precursors are homogeneously dispersed *via* dissolution in dichloromethane (DCM), followed by evaporation of the solvent to leave a homogeneously dispersed powder of the combined precursors which is subjected to thermal decomposition under an inert atmosphere. TGA of the individual precursors was measured to investigate the decomposition temperatures of the individual precursors. Figure S4 shows the individual (Figure S4a–g) and combined (Figure S4h) TGA plots of the precursors. From this analysis, all precursors were found to have decomposed to their subsequent metal sulfides at *ca*. 400 °C. This was therefore selected as the lowest temperature utilized in the decomposition of the homogeneously mixed precursor powder toward the synthesis of high entropy metal sulfide material. The decomposition temperature, time, and input precursor concentration were further optimized: the effect of these parameters is discussed in the Supporting Information, Figures S5–S7. Following this optimization, 500 °C was selected as the optimum temperature for a 1 h decomposition to produce multimetal containing high-entropy sulfides.

**Figure 2:**
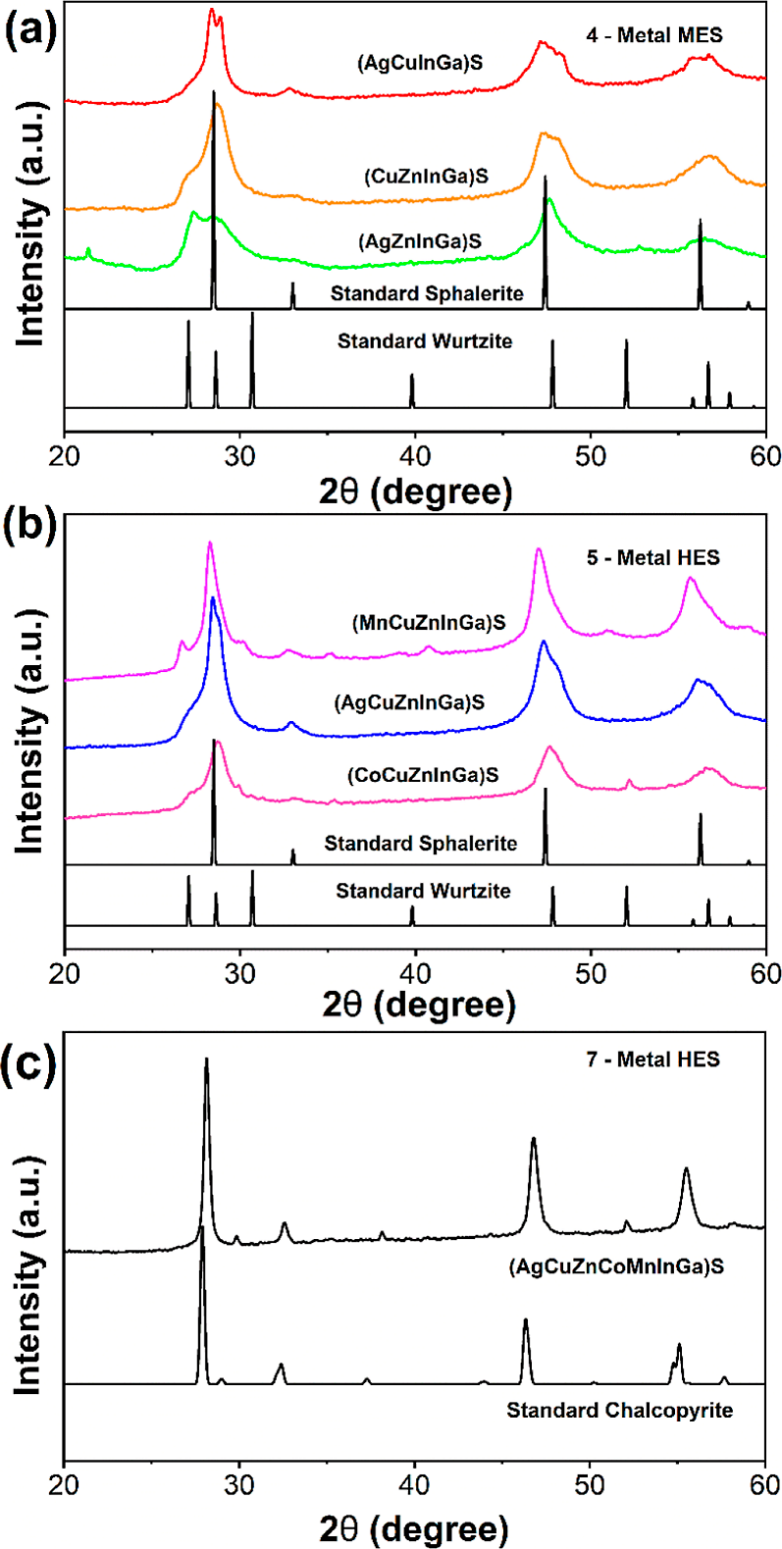
Powder XRD patterns of (a) the four-metal medium entropy systems (AgCuInGa)S, (CuZnInGa)S, and (AgZnInGa)S, (b) the five-metal HE systems (CuMnZnInGa)S, (AgCuZnInGa)S, and (CoCuZnInGa)S, and (c) the seven-metal HE system (AgCuZnCoMnInGa)S.

To demonstrate the versatility of this synthetic method, a range of four [(Cu, Zn, In, Ga), (Ag, Zn, In, Ga), and (Ag, Cu, In, Ga)], five [(Cu, Co, Zn, In, Ga), (Cu, Mn, Zn, In, Ga), and (Cu, Ag, Zn, In, Ga)], and seven (Cu, Ag, Zn, Mn, Co, In, Ga) dithiocarbamate precursors were combined in a stoichiometric molar ratio^a^ and decomposed, producing seven different composition multimetal sulfides. The phase purity of these seven produced materials was assessed by powder X-ray diffraction (pXRD). The subsequent patterns of all of the assessed materials are shown in Figure 2a–c. The four- and five-metal sulfides were all found to observe a dominant phase of sphalerite (ICSD: 34529), with some impurities of wurtzite (ICSD: 29474). The seven-metal sulfide was found to exhibit phase-pure chalcopyrite (ICDD: 2518), the crystal structure of the produced high entropy sphalerite and chalcopyrite are shown in Figure 1b,c, where the cationic positions are shown as multicolored spheres to represent the random orientation of each metal in the cationic lattice. It is important to note that HE materials have an inherent lattice distortion effect from the presence of multiple cations of various sizes in the cationic lattice positions. This effect will cause a shift in the 2θ reflections of the pXRD patterns compared to the standard patterns of the parent binary (sphalerite and wurtzite) and ternary (chalcopyrite) systems. To further demonstrate the purity of the entropy-stabilized (MnCoCuZnAgInGa)S, we undertook Rietveld refinements on the pXRD patterns of all seven synthesized systems. It was found that the fitting of (MnCoCuZnAgInGa)S was best performed with only chalcopyrite, further showing the phase purity of this system. The observation of a pure, entropically stabilized kinetic phase of HE metal sulfides is significant as these materials are said to be entropy stabilized and have demonstrated high performance and robustness in both thermoelectrics^19,20^ and electrocatalysis.^22,23^ To further analyze the morphology at both the micro- and nanoscale, along with the elemental distribution, energy-dispersive X-ray (EDX) spectroscopy was used in tandem with both SEM and STEM.

### SEM–EDX and STEM–EDX Analysis of the HE Metal Sulfides

**Figure 3:**
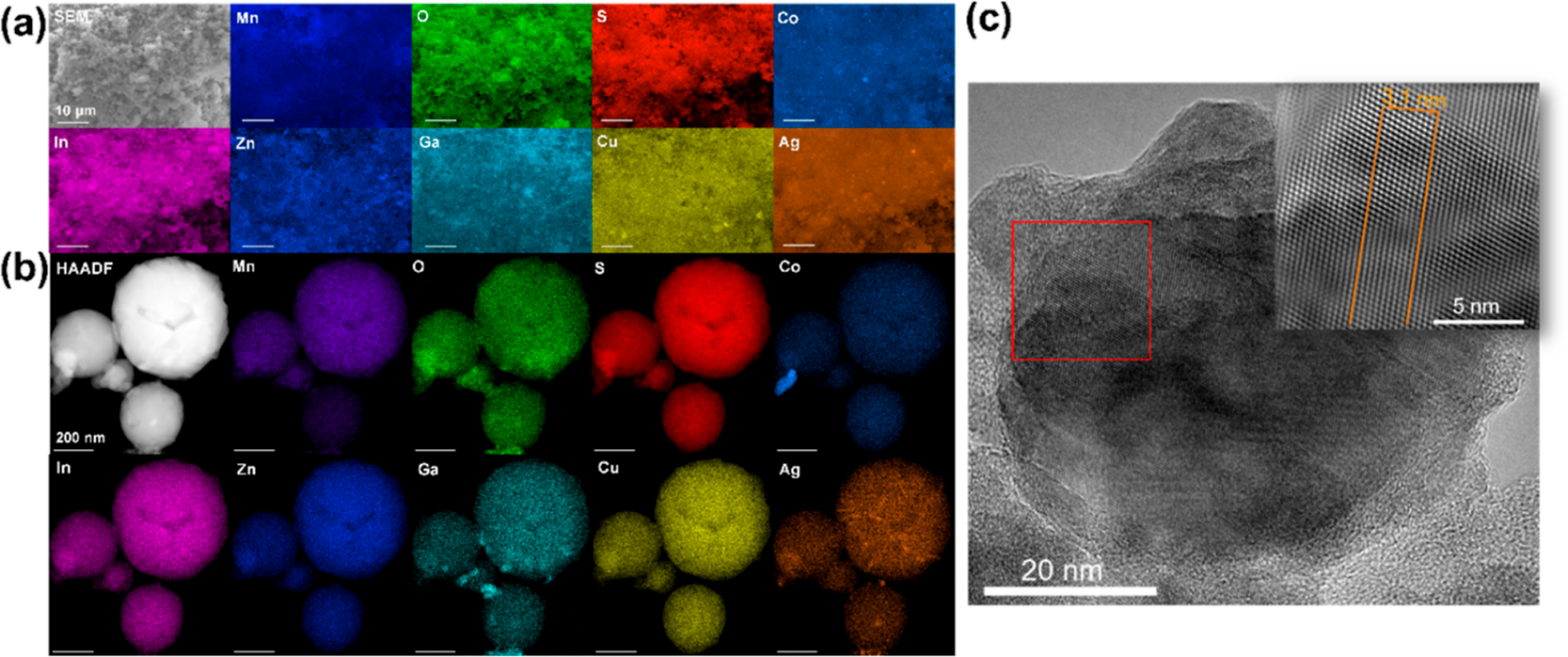
(a) SEM and EDX (20 kV) mapping of constituent elements of the (AgCuZnCoMnInGa)S material (scale bars represent 10 μm) and (b) TEM and EDX (200 kV) mapping of constituent elements of the (AgCuZnCoMnInGa)S material (scale bars represent 200 nm). (c) HR-TEM of (AgCuZnCoMnInGa)S with a lattice spacing of 3.1 Å (3.1 nm between 11 layers likely corresponding to the d_112_ lattice plane).

The morphology and elemental distribution of all HE metal sulfide materials produced were analyzed on the microscale by scanning electron microscopy (SEM) combined with EDX spectroscopy. The SEM–EDX maps of the four- and five-metal sulfide particulate materials are shown in Figures S11–S16. Figure 3a shows the SEM and EDX-spectroscopic mapping of the seven-metal sulfide HE material. This analysis shows a homogeneous dispersion of all elements throughout all produced HE materials at the microscale. The presence of oxygen was also observed, which we ascribe to surface oxidation during processing.

Having assessed the HE materials on the microscale, all seven produced materials were next investigated on the nanoscale using HAADF STEM withEDX spectroscopic mapping. Figure 3b shows the elemental distribution of all metals by STEM–EDX on the seven-metal HE material, with the four- and five-metal materials shown in Figures S17–S29. This analysis showed homogeneous dispersion of all elements on the nanoscale, with minor areas of what appear to be gallium oxide and potentially elemental silver (however, neither phase was observed by pXRD which suggests that these contaminatory phases are less than 1% w/w of the sample). The near-perfect distribution of these elements is a significant observation, as previous reports of HE metal chalcogenide materials have in part shown inhomogeneous distribution of metals at the nanoscale.^21,23,29^ The facile and low-temperature synthetic approach utilized in this report therefore represents a significant advance in the synthesis of bulk homogeneous HE metal sulfides. High-resolution TEM (HR-TEM) images in Figure 3c show an inter planar spacing of 3.1 Å (3.1 nm between 11 layers), which likely corresponds to the (112) lattice plane (*d*_112_ = 3.2 Å, 2θ = 28.1°) which is commensurate with pXRD data.

Quantitative information on the elemental composition of the synthesized materials can also be obtained from these analyses. Table S5 shows the elemental composition of the seven-metal HE material at both the micro- and nanoscale levels, along with surface analysis (using XPS, discussed below). This quantitative analysis shows that there is a slight discrepancy between the microscale and the nanoscale (discussed further in the Supporting Information).

### XPS of Seven-Metal Sulfide

**Figure 4:**
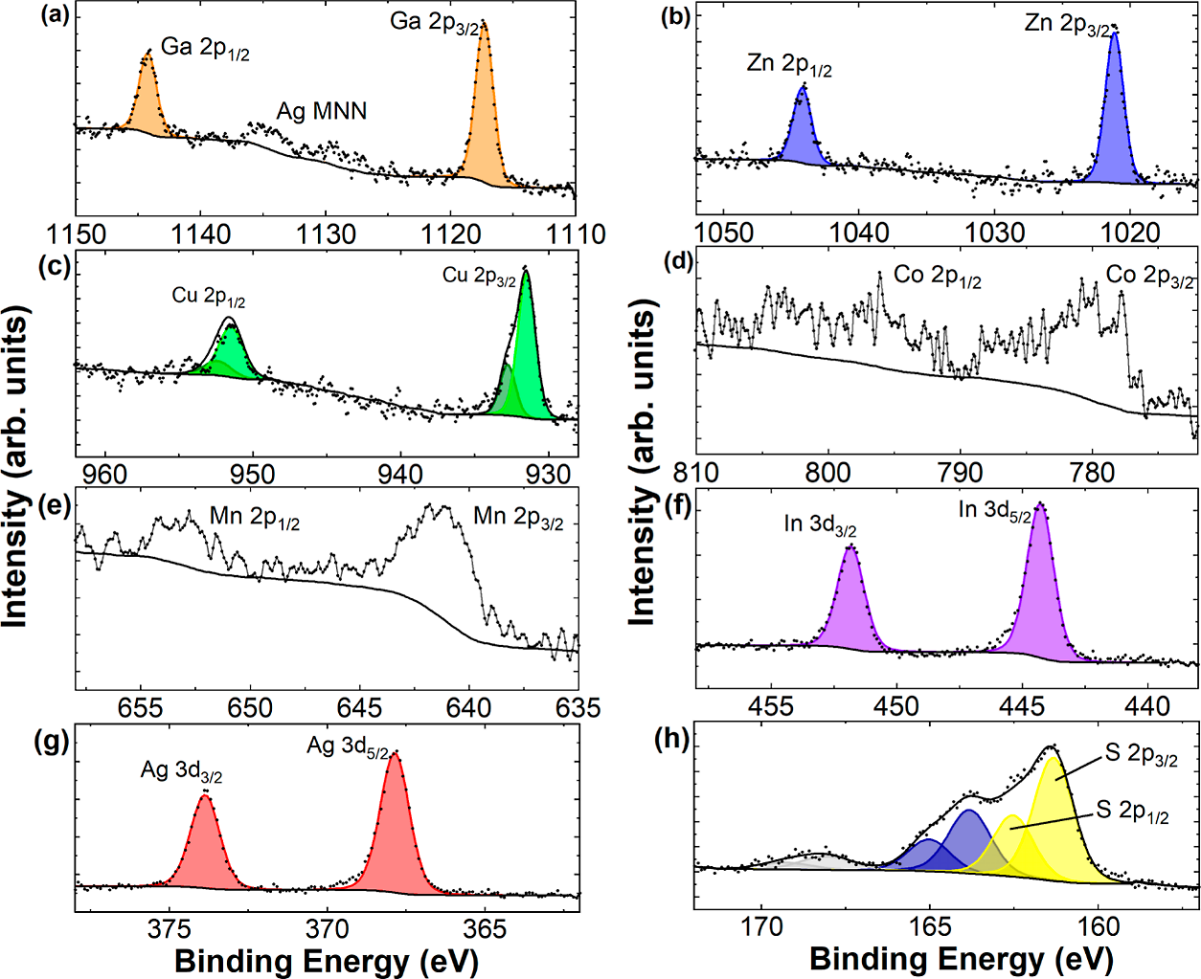
High-resolution spectra of (a) Ga 2p, (b) Zn 2p, (c) Cu 2p, (d) Co 2p, (e) Mn 2p, (f) In 3d, (g) Ag 3d, and (h) S 2p. Fitted peaks are shown in shaded color and discussed in the text; for Ga, Zn, Ag, and In, the spectra are well fit with one chemical species.

XPS was undertaken on the single-phased, seven-metal HE material to determine the valence state of the composition metals at the surface of the material (sampling depth for Al Kα is *ca*. 6 nm),^33^ (Figure S30 for the survey spectra). Initially, it could be determined that the ratio of metal/S is close to 1, but in excess of 1, which may indicate most of the metals are formed as MS, with some as M_*x*_S with *x* ≠ 1 (expected for a valence state ≠2). The chemical state of each metal was extracted by deconvoluting the peaks and extracting binding energy positions, as shown in Figure 4. For the case of Ag, In, Ga, and Zn, the peaks are well fit with one chemical species, with binding energy positions (with an estimated error of ±0.2 eV) of Ag 3d_5/2_ at 367.9 eV, In 3d_5/2_ at 444.4 eV, Ga 2p_3/2_ at 1117.6 eV, and Zn 2p_3/2_ at 1021.3 eV. These indicate silver is present as silver(I) (Ag_2_S),^34^ indium as In(III) (In_2_S_3_),^35^ gallium as Ga(III) (Ga_2_S_3_),^36^ and zinc as Zn(II) (ZnS)^37^ sulfides. Cu 2p indicates two chemical species, predominantly (>75%) as Cu_2_S with Cu 2p_3/2_ at 931.7 eV and Cu 2p_1/2_ at 951.6 eV binding energies.^38^ A second species is found with a peak position of 932.6 eV indicating copper oxide (Cu_2_O), and not sulfate (CuSO_4_) as this is expected at higher binding energies > 934.5 eV with additional satellite structure not evident in the spectra.^39^ Determination of Mn and Co is stymied somewhat by multiple splitting caused by paramagnetic interactions with unpaired d-electrons during photoemission, which is a facet that complicates the analysis of 2p photoelectron spectra from first row transition metals in general.^40^ The position of the Mn 2p_3/2_ peak at 641.2 eV is as expected comparing to literature on MnS^41^ and similarly the principle peak for Co 2p_3/2_ at 778.2 eV for cobalt sulfide.^42^ However, other peaks are also evident (e.g., some structure in Co 2p_3/2_ has a peak at 780.7 eV) again indicating a small amount of oxidation or potentially sulfate.^42^

The S 2p spectrum requires three chemical species (each a spin–orbit split doublet) in order to adequately fit the data, with S 2p_3/2_ binding energies of 161.3 (60% of the total intensity), 163.8 (31%), and 168.1 eV (9%). The latter is suggestive of sulfate and is a relatively small percentage (e.g., MnSO_4_,^43^). Given that most of the metals except Mn and Co show mainly one chemical species only, this sulfate may likely be more associated with these metals. The other S species may both be attributed to sulfide bonding with different metals (e.g., refs (44) and (45)), as is suggested by the close to unity ratio of total metal to sulfur (Table S6).

### Thermodynamic Analysis of the HE Materials

High entropy materials are so-called because the homogeneous mixing of multiple elements exhibits a large configurational entropy (*S*_conf_) In high entropy metal chalcogenides,^18^eq 1 is used to determine the configurational entropy of a material, from the number and fraction of mixed atoms
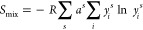
1where *S*_mix_ is the configurational entropy, *R* is the gas constant *a*^s^ represents the fraction of each sublattice (*S*) to the overall composition, and *y*_*i*_^s^ is the mole fraction of each constituent element (*i*) to the sublattice (*S*) it is contained within. Despite early reports into these materials stating that a value of 1.5*R* for the configurational entropy was required to achieve high entropy,^46^ we recently demonstrated that *S*_conf_ has been poorly estimated in many cases and this value cannot be achieved for a system with disorder in only one of the two sublattices, such as the cationic sublattice reported here.^18^ We therefore proposed high entropy metal chalcogenides with a single disordered sublattice were classified by the number of elements, with five or more elements being required in the disordered lattice.^18^

**Figure 5:**
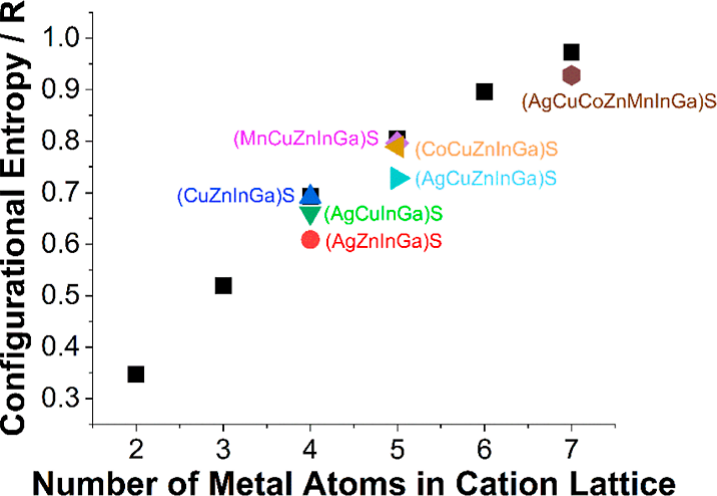
Calculated configurational entropy of a theoretical metal sulfide with an ideal molar ratio containing 1–7 metals [i.e., MS to (M^1^ M^2^ M^3^ M^4^ M^5^ M^6^ M^7^)S] (black squares) compared to our calculated HE materials containing four-, five-, and seven-metals (colored shapes all indicated with a label). Table of data can be found in Table S7.

For our synthesized systems, we therefore determine that we have synthesized three *medium entropy* systems (with four different metals in the cationic sublattice) and four *high entropy* materials (three with five metals and one with seven in the cationic sublattice). As the mole fraction of each element in our system can be quantified through EDX analysis, *S*_conf_ can also be calculated. The *S*_conf_ was initially calculated for an ideal one, two, three, four, five, six, and seven metal sulfide system, with the metals in an ideal equivalent molar ratio (Figure 5 black squares; tabulated in Table S7). The real *S*_conf_ for our seven synthesized materials was next calculated using the microscale elemental composition determined through SEM–EDX analysis. Figure 5 shows that our seven systems exhibit similar entropies of mixing than the ideal cases for the equivalent number of metals, demonstrating that our materials not only have excellent homogeneity of elements throughout the material but also have high *S*_conf_ in line with the expected values.

### Entropic Stabilization of Phases

From the pXRD analysis of the ME four- and HE five-metal sulfides (Figure 2), it is clear that there are two distinct phases present in these systems: cubic sphalerite and hexagonal wurtzite. For bulk sphalerite and wurtzite, sphalerite is marginally more stable in atmospheric pressure and room temperatures, with a free energy difference of ∼10 kJ mol^–1^.^47^ However, the entropy of wurtzite is ∼10 J mol^–1^ K^–1^ higher than that of sphalerite. Consequently, wurtzite and sphalerite are in equilibrium at 1020 °C and 1 bar (10^–4^ GPa)^47^ and wurtzite is more stable at higher temperatures. Using a combination of molecular dynamics simulations and thermodynamic analysis, it has been found that small ZnS nanoparticles in a vacuum are more thermodynamically stable as wurtzite over sphalerite. The transition temperature from nanocrystalline sphalerite to wurtzite decreases dramatically as the average particle size decreases below ∼20 nm.^48^ Given that the phase that is formed at higher temperatures is typically the more favorable phase (in this case wurtzite over sphalerite), the high configurational entropy governing our four- and five-metal sulfides is capable of stabilizing predominantly the metastable, cubic sphalerite phase. It, however, must be concluded here that the entropy alone of these systems is not high enough to stabilize a single phase entirely and as such the five-metal products of these reactions could be considered to be high entropy materials, rather than entropy-stabilized systems as a strict definition of the term.

In contrast, the HE seven-metal material (AgCuCoZnMnInGa)S was found to exist in a single phase i.e. tetragonal chalcopyrite. Tetragonal chalcopyrite is analogous to a double-stacked cubic sphalerite (Figure 1). It is significant that this new phase was observed without any crystalline impurities, which suggests that this is an example of a true entropy-stabilized system. The even higher configurational entropy observed in the HE seven-metal system was therefore able to stabilize phase-pure chalcopyrite.

HE metal chalcogenides are a new area of research and as such there is little literature reported to date.^18^ However, thorough research of the available literature of the reported phases achieved for HE metal chalcogenides showed that these HE materials typically favor a rock salt structure,^19,20,49,50^ although pentlandite^51^ and wurtzite^29^ have also been reported. A chalcopyrite HE metal chalcogenide has been reported with multimetal tellurides,^50^ one has been tentatively suggested with a multimetal sulfide but the extensive Scherrer broadening in the pXRD patterns stymied confirmation of this to any degree of accuracy. To date, no HE metal chalcogenide has reported a sphalerite structure. Therefore, here we can confirm the first reported HE metal sulfide with a sphalerite structure and the first confirmed HE metal sulfide with a chalcopyrite structure, both being examples of entropically stabilized systems.

### Electrocatalysis

Having successfully prepared an entropically stabilized phase-pure HE metal sulfide, we next set out to investigate this material for its catalytic ability to perform electrochemical HER from an acidic electrolytic environment (0.5 M H_2_SO_4_). To date, electrocatalysis using HE metal chalcogenides has been restricted to the oxygen evolution (OER)^21,23^ or CO_2_ reduction^22^ reactions, although in this limited scope, the clear advantages of using HE materials have been demonstrated for greater electrocatalytic performance. To explore the catalytic performance of our material for the HER process, an ink of the HE metal sulfide powder was prepared, drop-cast and dried on a glassy carbon electrode, and activated through cycling in an acidic solution (as described in the Supporting Information Experimental Section).

**Figure 6:**
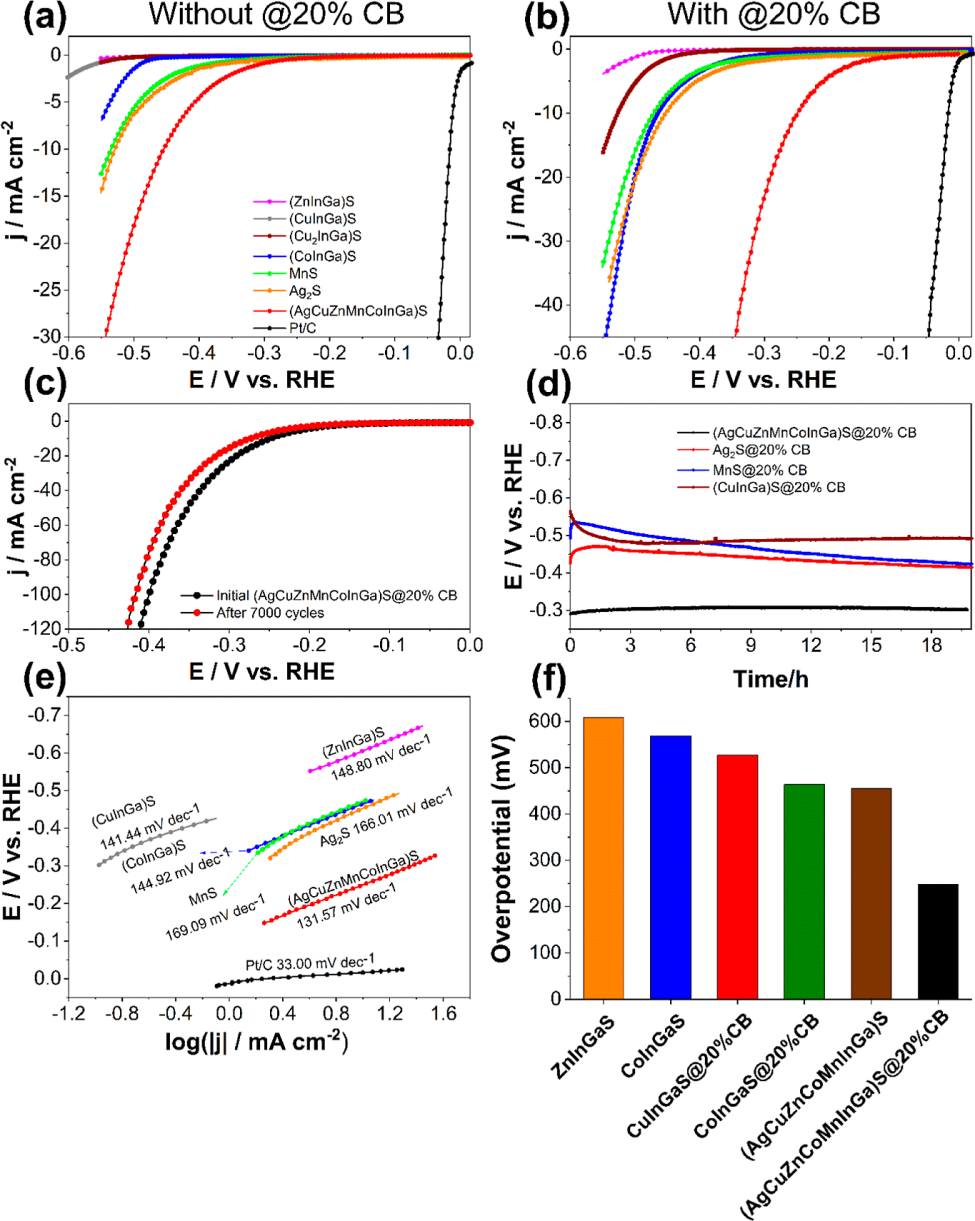
Electrochemical tests of as-prepared materials in 0.5 M H_2_SO_4_: (a,b) LSV within the HER potential range at a scan rate of 5 mV s^–1^ before and after the addition of 20% w/w of high conductivity carbon, respectively. (c) LSV curves of the HEMS@20% CB electrode before and after continuous cycling between −0.2 and −0.7 V vs Ag/AgCl for 7000 cycles at a scan rate of 200 mV s^–1^. (d) The durability test for 20 h at 10 mA cm^–2^ current density recorded using the chronopotentiometry technique (all areas are geometric surface area). (e) Tafel plots generated from the LSV curves of Figure (b). (f) η_10_ overpotential of all investigated materials in this report.

The catalytic ability of the material was determined through *iR*-compensated polarization curves recorded by using linear sweep voltammetry (LSV, Figure 6a). The onset overpotential (defined as the potential, relative to the thermodynamic value, where a current density of 1 mA cm^–2^ is attained) of (AgCuZnMnCoInGa)S was found to be −309 mV, with the corresponding overpotential to reach 10 mA cm^–2^ (η_10_) of −455 mV. It was expected that an increase in electrical conductivity of the system would improve performance. Therefore, to improve the electrical conductivity of the deposited ink, 20 wt % carbon black was added to yield (AgCuZnMnCoInGa)S@20% CB. The onset overpotential and η_10_ of this system were found to be significantly improved with the presence of carbon black (−80 and −255 mV, respectively, all data tabulated in Table S8). To draw direct comparisons in the performance of (AgCuZnMnCoInGa)S, phase-pure material was desired. For this reason, the synthesized four- and five-metal sulfides characterized here were not tested. We therefore prepared quaternary materials of sphalerite (CuInGa)S, (CoInGa)S, and (ZnInGa)S (pXRD patterns shown in Figure S31). The Ag and Mn analogues (AgInGa)S and (MnInGa)S were not synthesized due to the poor elemental solubility of Ag (Figure S7) and difficulty in obtaining high concentrations of Mn in the material (from the SEM–EDX). The (CuInGa)S, (CoInGa)S, and (ZnInGa)S systems were found to have onset overpotentials of −562, −435, and −441 mV and η_10_ of −569 mV for the (CoInGa)S system, respectively. The (CuInGa)S and (ZnInGa)S systems were not able to reach 10 mA cm^–2^, which is likely a conductivity issue of the material as measuring an η_10_ was possible with the addition of 20 wt % CB to the ink (Figure 6b). At overpotentials of −668 and −577 mV, the observed current densities were −8.32 and −0.46 mA cm^–2^, respectively, demonstrating the poor catalytic properties of the (ZnInGa)S system. These materials were also tested with 20 wt % carbon black, and (CuInGa)S@20% CB, (CoInGa)S@20% CB, and (ZnInGa)S@20% CB were found to have onset overpotentials of −435, −314, and −491 mV and η_10_ of −527, −464, and −608 mV, again demonstrating the significantly enhanced catalysis from the entropically stabilized chalcopyrite (AgCuZnMnCoInGa)S (all shown in Table 1). Metal monosulfides have been previously reported as HER electrocatalysts in acidic conditions, although the literature in this area is limited (Table S9). Cobalt-,^52–54^ silver-,^55,56^ and copper^57,58^-based sulfides have been reported and we compared the η_10_ to those found here. Co-based systems were found to yield η_10_ between 97 and 198 mV, Cu-based systems in the range of 86–193 mV, and Ag-based systems as 193–199 mV. However, these materials are nanoscale-designed materials such as nanoparticulate CuS@C,^57^ and hollow Cu/Cu_2_O/Cu_2_S nanotubes,^58^ nanoporous Ag_2_S/CuS composites,^55^ or CoNi_2_S_4_ nanorods, and nanoporous CuCo_2_S_4_ clusters.^54^ As electrocatalysis is typically reported in terms of geometric surface area, the high electroactive surface area yielded by these nanostructured materials will not be accurately accounted for and cannot be accurately normalized. Further reports have utilized doping such as the N-doped CoS, which yields further catalytic surface sites.^53^

**Table 1:** Data Showing the Onset and η_10_ for all Investigated Systems Both with and without 20 wt % Carbon Black

system	onset potential/mV	η10/mV	onset potential (20% CB)/mV	η_10_ (20% CB)/mV
MnS	396	535	305	470
Ag_2_S	382	529	287	452
(CuInGa)S	562	/	435	527
(ZnInGa)S	435	/	314	464
(CoInGa)S	441	569	491	608
(AgCuZnMnCoInGa)S	309	455	80	255

To investigate more fundamental properties of our electrocatalysis, the Tafel slope values were determined from our carbon-black enhanced systems (Figure 6b). This provides insights into the overall mechanistic aspects of the HER on our systems. All Tafel slope values were found to be within the range of 130 and 150 mV dec^–1^, with the (AgCuZnMnCoInGa)S@20% CB having the lowest (131.6 mV dec^–1^) and the (ZnInGa)S the highest (148.8 mV dec^–1^). This range of values suggests that HER proceeds *via* the Volmer mechanism, according to the general models of HER.^59^ EIS was also employed to obtain further quantitative information about fundamental electrocatalysis (Figure S33). EIS showed that the charge transfer resistance (*R*_ct_) was also lower in the seven-metal system (4.98 Ω cm^2^) than the other synthesized quaternary materials with (CoInGa)S, (CuInGa)S, and (ZnInGa)S yielding an *R*_ct_ of 7.39, 8.59, and 9.45 Ω cm^2^, respectively. Previous reports of metal sulfides that have been utilized for HER has shown a range of *R*_ct_ values between 1.58 and 71 Ω, our absolute *R*_ct_ values were found to be in the range of 70.4–133.6 Ω,^b^ comparing favorably to the literature reported values (Table S9 shows the comparison data and references). This also shows that the lower η_10_ observed from previously reported metal sulfides for HER stems from the significantly enhanced surface area from the designed nanostructuring. Again, this analysis showed the significant benefit of the entropically stabilized (AgCuZnMnCoInGa)S material.

Further insights into these systems can be obtained by determining the approximate electrochemically active surface area. This was achieved by measuring *V*_s_ of restricted windows at various scan rates and determining the double layer capacitance (*C*_dl_) as a *pseudo*-surface area,^60^ as the latter is directly proportional to the former^61^ (Figure S34). As expected for a blocking interface, current density is proportional to scan rate, and thus *C*_dl_ can be determined by the slope of these curves. *C*_dl_ of our investigated systems were found to be 2.40, 1.06, 3.10, and 1.46 mF cm^–2^ for the (AgCuZnMnCoInGa)S@20% CB, (CoInGa)S@20% CB, (CuInGa)S@20% CB, and (ZnInGa)S@20% CB, respectively. The high *C*_dl_ for the (CuInGa)S@20% CB system is likely due to overlapping Faradaic processes, which are likely to be a redox process of Cu and subsequent leaching from the material. This system was therefore discounted from further analysis. The contribution from CB alone was determined to be 0.22 mF cm^–2^ and was subtracted from the values. By combining impedance (*R*_ct_) and surface area (*C*_dl_) effects it is possible to deconvolute the contribution of surface area alone to catalytic activity (analogous to *j* normalized to ECSA).^61^ The *R*_ct_*C*_dl_ was therefore calculated and determined as 10.86, 6.21, and 11.72 ms for (AgCuZnMnCoInGa)S@20% CB, (CoInGa)S@20% CB, and (ZnInGa)S@20% CB, respectively. This analysis shows that despite the (ZnInGa)S@20% CB having a lower surface area than (AgCuZnMnCoInGa)S@20% CB, it has roughly equivalent intrinsic activity, while the (CoInGa)S@20% CB has roughly double the intrinsic activity of the other two systems. We also compared the current density (per nominal area) ratios of the four systems at an overpotential of −0.4 V versus RHE. (AgCuZnMnCoInGa)S@20% CB was found to have an increased current density (at −0.4 V) of 28×, 228×, and 627×, compared to the (CoInGa)S@20% CB, (CuInGa)S@20% CB, and (ZnInGa)S@20% CB, respectively. These values of increased current density are significantly larger than the comparative increase of *C*_dl_ (2.26, 0.77, and 1.64), and *R*_ct_*C*_dl_ (1.75, 0.44, and 0.93) of (AgCuZnMnCoInGa)S@20% CB, compared to the (CoInGa)S@20% CB, (CuInGa)S@20% CB, and (ZnInGa)S@20% CB systems, respectively. This analysis highlights that the intrinsic properties of the entropy-stabilized (AgCuZnMnCoInGa)S@20% CB are the major contributing cause of the high electrocatalysis performance and the difference in surface area is only a minor contribution to the changes in properties seen.

Finally, the medium- and long-term stability of the entropy-stabilized (AgCuZnMnCoInGa)S@20% CB was assessed. Medium-term stability was assessed by cycling the potential from −0.2 to −0.7 V versus Ag/AgCl at a scan rate of 200 mV s^–1^ for 7000 cycles (Figure 6c). This analysis showed that over 7000 cycles, the η_10_ increased by only 25 mV, indicating excellent stability of (AgCuZnMnCoInGa)S@20% CB. Long-term stability was assessed by applying a constant current of 10 mA cm^–2^ for 20 h and is shown in Figure 6d. We also tested the long-term stability of chalcopyrite (Cu_2_InGa)S and binary MnS and Ag_2_S as a direct comparison. This showed that over a 20 h period, the overpotential required to maintain 10 mA cm^–2^ with the (AgCuZnMnCoInGa)S@20% CB system remained at 300 ± 10 mV, again demonstrating excellent stability. In contrast, the binary and quaternary systems exhibit notable fluctuations in overpotential within a similar time frame, indicating that the entropy stabilization achieved through the interactions between the different incorporated elements in (AgCuZnMnCoInGa)S@20% CB sample exhibiting superior structural stability.^62,63^ Comparisons of our long time study with similar studies of other reported metal sulfides can be found in Table S10. It is important to note that, catalysis is highly dependent on system architecture, fortuitous or tailored surface defects, material dopants and true electrochemically active surface area.^64–67^ We have demonstrated that our entropy-stabilized (AgCuZnMnCoInGa)S material significantly outperforms our other tested systems. This is without any optimization of the aforementioned properties, which would be expected to yield considerable increases in performance. Another potential area for optimization in these entropy-stabilized materials from the precursor synthesis route is the ability to utilize transition, main group, and lanthanide metals, potentially unlocking limitless combinations available for design and optimization toward catalysis.^32^ Additionally, computational analysis of various metals within the lattice toward electron transfer to hydrogen could lead to significant further development of performance in these systems.

## Conclusions

In summary, we have demonstrated a scalable, facile, rapid (1 h), and low-temperature (500 °C) approach toward the synthesis of HE metal sulfides *via* the simultaneous decomposition of multiple metal dithiocarbamate single source precursors. The versatility of this approach has been demonstrated by the successful synthesis of four, five, and seven metal containing HE materials. The synthesized HE materials were all characterized by SEM and TEM, both with EDX spectroscopy to investigate the composition and distribution of the metals throughout the materials. It was found that this approach yielded even distributions of metal cations throughout the material at both the micro- and nanoscales, which is an advantage over other approaches of synthesizing high entropy metal sulfides such as elemental annealing, that found localized metal clustering on the nanoscale. The obtained phase of these materials was also examined by pXRD, which found that for the four and five metal containing materials a dominant phase of sphalerite was observed, with some wurtzite impurities. The seven metal HE material was found to observe phase-pure chalcopyrite material, which was facilitated by the high configurational entropy of the extra metals within the material. The electrocatalytic properties of the materials with respect to the hydrogen evolution reaction was also tested. The (AgCuZnMnCoInGa)S@20% CB was found to have a low onset potential (∼80 mV) and η_10_ (∼255 mV). This comfortably out-performed other synthesized metal sulfides, where the high performance is caused by the high entropy nature of the material. It is also expected that this scalable single source precursor approach can be extended and will be universal to other transition and main group metals due to the extensive library of metal dithiocarbamate precursors available.

## Supporting Information Available

Chemicals and methods, CVs, pXRD pattern, IR spectra, Rietveld refinement data, SEM–EDX images, STEM–EDX images, XPS spectra, impedance and surface area data, and comparisons to literature data (DOCX)

## Footnotes

a With exception for Ag, which was found to have a low solubility in the high entropy material (Figure S7) and was therefore lowered to 0.25 stoichiometric molar ratio.

b Note here that our *R*_ct_ values are reported in Ω cm^2^; however, for the comparison with the literature, only absolute resistance (not areal resistance) was found. So, we therefore converted our areal resistance to absolute resistance in order to draw this comparison.
