# Supplementary material for: Synthesis of High Entropy and Entropy-Stabilized Metal Sulfides and Their Evaluation as Hydrogen Evolution Electrocatalysts
Source: Chem Mater. 2023 Sep 19;35(19):7904–14. doi: 10.1021/acs.chemmater.3c00363 (PMC10568966; doi:10.1021/acs.chemmater.3c00363)
Supplement: Supplementary file 1 — cm3c00363_si_001.docx [file cm3c00363_si_001.docx]

Supporting Information for:

Facile Synthesis of High Entropy and Entropy-Stabilised Metal Sulfides and their Evaluation as Hydrogen Evolution Electrocatalysts

Weichen Xiao,^a,†^ Yi Li,^a,†^ Amr Elgendy,^b,c^ Ercin C. Duran,^a^ Mark A. Buckingham,^a^ Ben F. Spencer,^a^ Bing Han,^a^ Firoz Alam,^a^ Xiangli Zhong,^a^ Sarah H. Cartmell,^a^ Robert J. Cernik,^a^ Alexander S. Eggeman,^a^ Robert A. W. Dryfe,^b^ and David J. Lewis^a,*^

^a^ Department of Materials, The University of Manchester, Manchester M13 9PL, U.K.

^b^ Department of Chemistry, The University of Manchester, Oxford Road, Manchester, M13 9PL, UK.

^c^Egyptian Petroleum Research Institute, 11727 Cairo, Egypt.

† These authors contributed equally to this work

* Corresponding author: [david.lewis-4@manchester.ac.uk](mailto:david.lewis-4@manchester.ac.uk)

# Contents

**Chemicals and Methods**

**Figure S1** – Cyclic voltammograms of pure and impure Co(DTC)_3_

**Figure S2** – pXRD patterns of (MnCoCuZnAgInGa)S synthesied with Mn(DTC)_3_ and Co(DTC)_3_ compared to Mn_2_O_2_(DTC)_4_ and ‘Co(DTC)_3_’

**Table S1** – Table of elemental composition from XPS analysis

**Figure S3** – IR analysis of precursors

**Figure S4** – Temperature analysis of synthesis of HE materials

**Figure S5** – pXRD for temperature analysis on the synthesis of HE materials

**Figure S6** – pXRD for time analysis on the synthesis of HE materials

**Figure S7** – pXRD analysis of Ag^+^-containing HE materials

**Figure S8** – 500 °C *vs* 1000 °C synthesis of (MnCoCuZnAgInGa)S

**Figure S9, S10** – Rietveld refinements of pXRD patterns

**Table S2 – S4** – Table of data for Rietveld refinements

**Figure S11 – S16** – SEM-EDX maps of the four and five metal containing HE materials

**Figure S17 – S29** – STEM-EDX maps and line scans of the four and five metal containing HE materials

**Table S5** – Table of data quantitatively comparing element composition of the seven metal HE material from SEM-EDX, STEM-EDX, and XPS analysis

**Figure S30** – Survey spectra for XPS analysis

**Table S6** – Metal : Sulfur ratio from XPS analysis

**Table S7** – Table of data of configuration entropy calculated for synthesised HE materials

**Figure S31, 32** – pXRD of the (CuInGa)S, (CoInGa)S, (ZnInGa)S, (Cu_2_InGaS)

**Figure S33** – Electrochemical impedance spectroscopic analysis

**Figure S34** – Electrochemical surface area analysis data

**Table S8** – Data table for electrocatalysis

**Table S9** – Table of data compared to reported metal sulfide HER electrocatalysis

**Table S10** – Table of data for long-time studies compared to reported metal sulfide HER electrocatalysis

# Methods

## Chemicals

All chemicals were purchased from UK suppliers and used without purification, unless otherwise specified. Sodium diethyldithiocarbamate trihydrate (97%, Sigma Aldrich), silver diethyldithiocarbamate (99%, Sigma Aldrich), copper(II) chloride (CuCl_2_ 99%, Sigma Aldrich), zinc (II) chloride (ZnCl_2_ 98%, Sigma Aldrich), cobalt chloride (CoCl_2_ 98%, Sigma Aldrich), gallium(III) nitrate hydrate (Ga(NO_3_)_3_ 99.9%, Sigma Aldrich) indium(III) chloride (InCl_3_ 99.999%, Sigma Aldrich) and manganese(II) acetate tetrahydrate (Mn(CH_3_CO_2_)_2_.4 H_2_O ≥99% Sigma Aldrich).

## Synthesis

Synthesis of single source precursors was undertaken using a facile metathesis reaction of metal salt and sodium diethyldithiocarbamate. All syntheses were performed in atmospheric conditions, no special handling or inert conditions was required and are outlined in detail in the supporting information. IR spectroscopy was undertaken on all synthesised precursors and spectra are presented in Figure S4.

## Synthesis of copper dithiocarbamate, Cu(S_2_CNEt_2_)_2_

Cu(S_2_CNEt_2_)_2_ was synthesised following a literature procedure,^1^ briefly: sodium diethydithiocarbamate trihidrate (1.35 g, 6 mmol) was dissolved in methanol (30 mL). Copper chloride (0.40 g, 3 mmol) was dissolved in a separate solution of methanol (20 mL). The copper chloride solution was added dropwise to the diethyldithiocarbamate solution. Once fully added, the mixture was stirred for one hour at room temperature. After one hour had elapsed, the product was collected by vacuum filtration, washed with methanol and DI water at room temperature and dried under vacuum overnight. The final product was a dark brown solid, with a yield of 86.4%. CHN Anal. Calc for C_10_H_20_S_4_N_2_Cu. Expected: C 33.4%, H 5.6%, N 7.8%, S 35.6%. Found: C: 33.5%, H: 5.6%, N: 7.7%, S: 35.6%.

## Synthesis of zinc dithiocarbamate, Zn(S_2_CNEt_2_)_2_

Zn(S_2_CNEt_2_)_2_ was prepared using the same method as Cu(S_2_CNEt_2_)_2_, where zinc chloride (0.41g 3 mmol) was the zinc source. The final product was a white solid, with a yield of 83.9%.

CHN Anal. Calc for C_10_H_20_S_4_N_2_Zn. Expected: C: 33.2%, H: 5.6%, N: 7.7%, S: 35.4%. Found: C: 33.7%, H: 5.6%, N: 7.7%, S: 35.6%.

## Synthesis of indium dithiocarbamate, In(S_2_CNEt_2_)_3_

In(S_2_CNEt_2_)_3_ was synthesised by the same method as Cu(S_2_CNEt_2_)_2_, where indium chloride (0.66g, 3 mmol) was the indium source. The only difference was that the higher amount of sodium diethydithiocarbamate trihidrate (2.03g, 9 mmol) required. The final product was a white solid. Yield 84.5%. CHN Anal. Calc for C_15_H_30_S_6_N_3_In. Expected: C: 32.2%, H: 5.4%, N: 7.5%, S: 34.4%. Found: C: 32.5%, H: 5.4%, N: 7.5%, S: 34.5%.

## Synthesis of gallium dithiocarbamate, Ga(S_2_CNEt_2_)_3_

Ga(S_2_CNEt_2_)_3_ were prepared using the same method as In(S_2_CNEt_2_)_3_, where gallium nitrate (0.78g, 3 mmol) was used as gallium source. The final product was a white solid with a yield of 85.2%. CHN Anal. Calc for C_15_H_30_S_6_N_3_Ga. Expected: C: 35.0%, H: 5.9%, N: 8.2%, S: 37.4%. Found: C: 33.9%, H: 6.6%, N: 7.9%, S: 34.4%.

## Synthesis of cobalt dithiocarbamate, Co(S_2_CNEt_2_)_2_ and Mn(S_2_CNEt_2_)_2_

Synthesis of both Co and Mn diethyldithiocarbamate (DTC) were initially undertaken using 2 equivalents of DTC : metal, the elemental analysis for both of these came back pure and were thus used in all further investigations. However, during preparation of the manuscript we discovered these precursors were susceptible to oxidation and we therefore conducted further characterisation of these species as below.

## A note on oxidation Mn and Co precursors

The Mn and Co precursors were initially synthesised using 2 equivalents of diethyldithiocarbamate ligands to balance the charge of the starting Mn and Co salts. The product was analysed with elemental analysis and were both found to fit with the anticipated Mn(DTC)_2_ and Co(DTC)_2_ products. However, these species rapidly oxidise to the Mn(DTC)_3_ and Co(DTC)_3_.^2-4^ synthesis methods have been reported which use the Mn(DTC)_2_ and Co(DTC)_2_, but we would like to point out that these have been wrongly reported and are likely to be the Mn(DTC)_3_ and Co(DTC)_3_ species in fact.^4^ In our study, elemental analysis was found to fit the Mn(DTC)_2_ and Co(DTC)_2_ species, which did not align with literature, in particular the studies by Eagle *et*. *al*. and Hendrickson *et*. *al*.^2, 3^ This prompted further investigation into the exact complexes which we had made. The magnetic susceptibility ($\mu_{eff}$) was measured for both species. The ‘Mn(DTC)_2_’ and ‘Co(DTC)_2_’ were found to have a $\mu_{eff}$ of 3.76 BM and 2.36 BM, respectively. These values are far from the expected value for tetragonal Mn(DTC)_2_ of 1.76 BM and 5.92 BM for low spin and high spin complexes, respectively, and square planar Co(DTC)_2_ of 1.73 BM and 3.87 BM for low spin and high spin complexes, respectively, indicating these are not the species present.

The ‘Mn(DTC)_2_’ precursor is in reality a dimeric [O_2_]^2-^-bridging species (Mn_2_O_2_(DTC)_4_) that we have determined and recently reported.^5^ The ‘Co(DTC)_2_’ has not yet been assessed so we therefore investigated this system further here. Cyclic voltammograms were recorded,^[[1]](#footnote-2)^ of the ‘Co(DTC)_2_’ and compared to latterly synthesised pure Co(DTC)_3_, synthesised with 3 equivalent of DTC ligand, which also included a purification step (preparation and characterisation outlined below). It was found that both Co species had the same voltametric response, indicating that equivalent species were present in solution from both synthetic procedures (Figure S1). The concentration normalised current density of the ‘Co(DTC)_2_’ was reduced when compared to the Co(DTC)_3_, which shows the impure nature of the original ‘Co(DTC)_2_’ precursor. Crystals were grown of both the 2 and 3 equivalent DTC synthesised materials and the structures solved, with both found to be the [Co(DTC)_3_], consistent with a structure previously reported (CCDC number: 1150428).

**Figure S1**. Figure showing (a) the cyclic voltametric response of the cobalt diethyldithiocarbamate complexes synthesised with 2 eq. DTC (purple) and 3 eq. DTC (green). These were recorded on a glassy carbon electrode in acetone at 2.5 mM concentrations with 0.1 M [TBA][PF_6_] supporting electrolyte at 100 mV s^-1^ scan rate and a Ag/AgCl reference electrode.

With the precursors fully identified and characterised, we used the pure [Mn(DTC)_3_] and [Co(DTC)_3_] precursors in the synthesis of further HE 7-metal (MnCoCuZnAgInGa)S to show that equivalent material can be synthesised with [Mn(DTC)_3_] and [Co(DTC)_3_] as to that synthesised with pure Mn_2_O_2_(DTC)_4_ and impure ‘Co(DTC)_2_’, which was used in all further investigations. Powder XRD of the (MnCoCuZnAgInGa)S synthesised with the [Mn(DTC)_3_] and [Co(DTC)_3_] precursors show that equivalent bulk material was made and directly comparable to the sample used in this report (Figure S2).


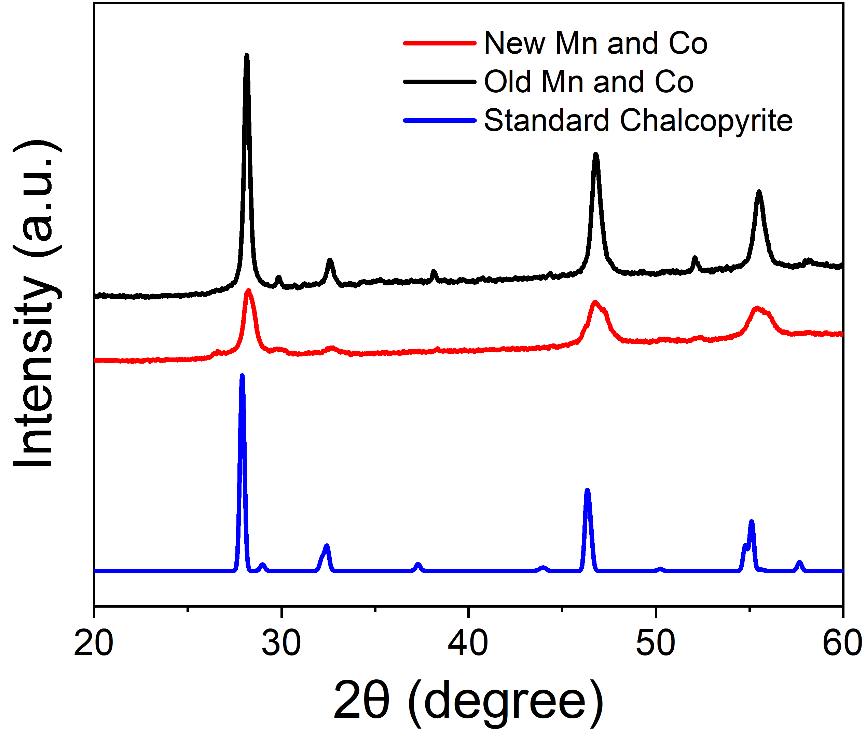


**Figure S2**. Powder X-ray diffraction pattern of synthesised (MnCoCuZnAgInGa)S with the Mn_2_O_2_(DTC)_4_ and ‘Co(DTC)_2_’ (black) and with pure Mn(DTC)_3_ and Co(DTC)_3_ (red).

## Synthesis of pure Mn(diethydithiocarbamate)_3_ ([Mn(DTC)_3_])

Synthesis of [Mn(DTC)_3_] was undertaken following an adapted previous procedure.^6^ Separately, MnCl_2_ (1.98 g, 10 mmol) was dissolved in 50 mL of methanol and NaDTC (6.76 g, 30 mmol) was dissolved in 150 mL of methanol, both with stirring. The solution of MnCl_2_ was gradually added to the solution of NaDTC while under constant stirring (note that the Mn(ii) readily oxidises to Mn(iii) upon formation of Mn(DTC)_2_.^4^ This was left for 2 hours, and the resultant precipitate collected under vacuum filtration, analysis of this product was found to be impure so a purification step was required by dissolving all impure material in 20 mL of dichloromethane with stirring for a short period, followed by the rapid addition of 10 mL ethanol. Stirring was continued for a couple of minutes, followed by a period of rest without stirring for an equivalent amount of time. The black filtrate was dried under vacuum overnight and the resultant dry powder used in subsequent investigations. Elemental analysis found (expected for Mn(C_15_H_30_N_3_S_6_) $\cdot$ 0.5 H_2_O. C: 35.3 (35.4), H: 6.4 (5.9), N: 8.1 (8.3), S: 37.1 (37.7). IR (cm^-1^): 571, 596, 782, 844, 912, 988, 1069, 1094, 1143, 1205, 1266, 1295, 1348, 1377, 1424, 1440, 1457, 1487, 1509, 2865, 2926, 2971. m/z: 499.0. μ (μ_eff_): 5.07 (4.90) BM.

## Synthesis of pure Co(diethyldithiocarbamate)_2_ ([Co(DTC)_3_])

Synthesis of [Co(DTC)_3_] was undertaken following the same adapted procedure.^6^ Separately, CoCl_2_ (2.30 g, 10 mmol) was dissolved in 50 mL of methanol and the NaDTC (6.76 g, 30 mmol) was dissolved in 150 mL of methanol, both with stirring. The solution of CoCl_2_ was gradually added to the solution of NaDTC while under constant stirring. This was left for 2 hours, and the resultant precipitate collected under vacuum filtration. The dark green filtrate was dried under vacuum overnight and the resultant dry powder used in subsequent investigations. Elemental analysis found (expected for Co(C_15_H_30_N_3_S_6_) $\cdot$ 0.5 H_2_O. C: 34.8 (35.1), H: 5.9 (5.9), N: 8.1 (8.2), S: 37.2 (37.4). IR (cm^‑1^): 564, 581, 602, 784, 846, 913, 996, 1059, 1074, 1132, 1147, 1211, 1263, 1292, 1351, 1373, 1431, 1449, 1482, 2865, 2926, 2971, m/z (+Na): 526.00. μ (diamagnetic).

## X-ray photoelectron spectroscopy (XPS)

X-ray Photoelectron Spectroscopy (XPS) was performed using an Axis Ultra Hybrid spectrometer (Kratos Analytical, Manchester, United Kingdom) using monochromated Al Kα radiation (1486.6 eV, 10 mA emission at 150 W, spot size 300 $\times$ 700 μm) with a base vacuum pressure of ~5 $\times$10^-9^ mbar. Powdered material was pressed onto conductive tape, and charge neutralisation was used to remove any differential charging effects. The X-ray beam size w\s 300 $\times$ 700 μm. Binding energy scale calibration was performed using C-C in the C 1s photoelectron peak at 284.8 eV. Analysis and curve fitting was performed using Voigt-approximation peaks using CasaXPS.

Two positions on the powdered material were measured, and errors on atomic concentrations calculated as a combination of the standard deviation of extracted photoelectron intensities and the sensitivity to the elemental core level (related to the relative sensitivity factor of the core level). The average atomic concentration for the elements is listed in Table S1. Carbon and oxygen are likely to be mainly attributed to C and C-O type contamination at the surface, which is often enhance by a greater surface area in powdered materials.

**Table S1** - Atomic concentrations calculated from two positions on the powdered material. These calculations suggest that atomic ratios of Co: Cu: Ga: Mn: Zn are close to 1:1, with Ag:In also close to 1:1 but in excess of 3 times greater than the other metals. This ratio obtained after the removal of C and O.

| Element | Ag | Co | Cu | Ga | In | Mn | Zn | S |
| --- | --- | --- | --- | --- | --- | --- | --- | --- |
| Atomic % | 16.77 | 2.84 | 4.88 | 5.94 | 13.75 | 3.02 | 3.91 | 48.89 |
| Error % | 0.35 | 0.62 | 0.62 | 0.27 | 0.35 | 0.44 | 0.35 | 2.93 |

## Electrochemistry

The electrochemical workstation (Metrohm Auto-Lab potentiostat PGSTAT302N, equipped with the FRA32 module) with standard three electrode configuration was used to carry the electrochemical activity for HER in 0.5 M H_2_SO_4_ purged with N_2_. A two-compartment cell configuration with a working and reference electrodes separated from a Pt mesh counter electrode by a poly(vinyldifluoridene) membrane with pores of 100 nm diameter. With this setup, contamination from the counter electrode is avoided, as previously reported in the literature.^7, 8^ The catalyst suspension was carried out by mixing the active material and carbon black Super P (Alfa Aesar, 99+% metals basis) in the ratios required to reach 0, and 20 wt%. The mixture was ultrasonically dispersed in a water-ethanol solution (v/v=4:1) containing 0.04 wt.% Nafion^®^ solution (Sigma Aldrich, protonic form, 5 % w/w solution in a mixture of lower aliphatic alcohols and 45% water). the resultant ink was drop cast on 3 mm diameter glassy carbon with catalyst loading of 0.6 mg/cm^2^. Potentials were referenced to the reversible hydrogen electrode (RHE): E(RHE) = E(Ag/AgCl) + (0.205 + 0.059 pH) V. where, the final potential was converted into RHE by adding a value of 0.222V.

The electrocatalytic performance of the prepared materials towards HER was investigated *via* custom-made experimental procedures integrated with Nova software, similar to what was reported previously.^9, 10^ Prior to any measurement, the working electrodes were cycled at least 2000 times using a cyclic voltammetry (triangular) waveform at 500 mV s^-1^. This was done to activate the entire surface of the catalyst material by removing any trapped air within the catalyst during the preparation process. Linear-sweep voltammetry (LSV) was recorded at scan rate of 5 mV s^-1^ to obtain the polarisation curve. The Tafel slope was calculated for assessing the HER kinetics of the catalyst by the Tafel equation (η = a+ blog(*j*), where η, *j*, and b represent the overpotential, current density, and Tafel slope, respectively). All the data presented were corrected for i*R* losses and background current. EIS measurements were recorded in the frequency range of 20 kHz to 100 mHz. with an amplitude of 7 mV peak-to-peak. All EIS data were subjected to a Kramers-Kroning (K-K) test. Only the data which complied with the K-K criteria (*i.e.*, providing relative residuals of less than 5% for both the real and imaginary parts of the impedance) was used for the EIS analysis. The moduli of the calculated impedances were used as weighting factors. The chi-square parameter, indicative of the goodness of the fit, was in the order of 10^-3^ for all data. The electrochemical active surface area (ECSA) was estimated by calculating the double layer capacitance of the electrodes from cyclic voltammetry data to various scan rates from 20 to 100 mV s^-1^ in the potential region of 0.02 – 0.18 V (vs. RHE). The medium-term stability of the prepared material was evaluated by continuous potential cycling in the potential window between −0.2 and −0.7 V vs. Ag/AgCl_(3.5 M KCl)_ at a scan rate of 200 mV s^-1^ for 7000 cycles. Polarization curves were recorded, based on the approach described above, before and after the CV experiment, and the obtained data are compared. For the long-term stability test, the chronopotentiometry test was conducted at 10 mA cm^-2^ for 20 h under stirring to decrease the bubble formation over the electrode surface within the experimental timeframe.

# Infra-Red (IR) spectroscopy of synthesised precursors


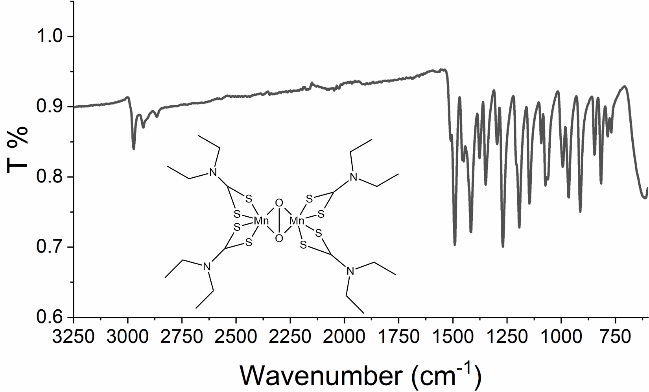

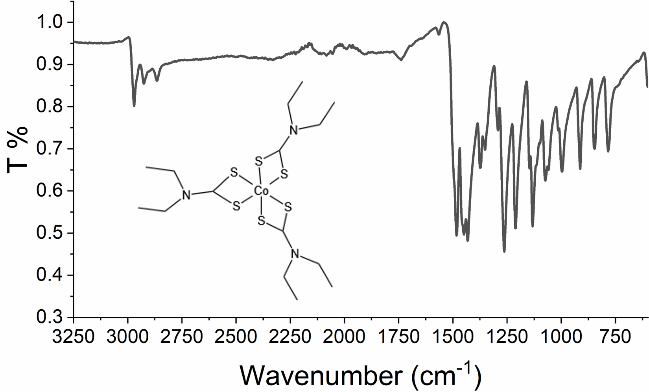

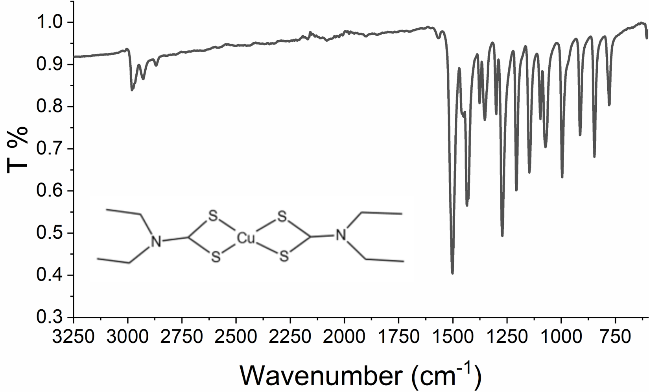

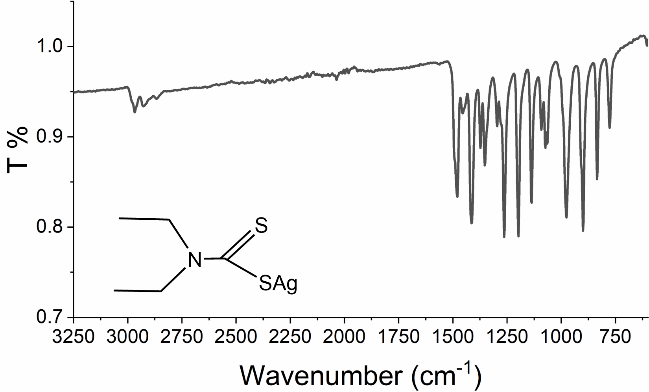

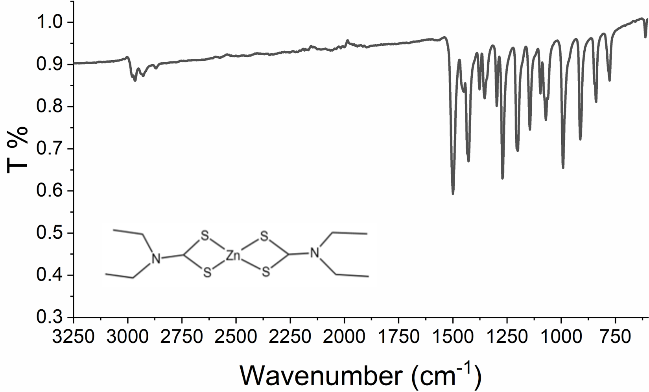

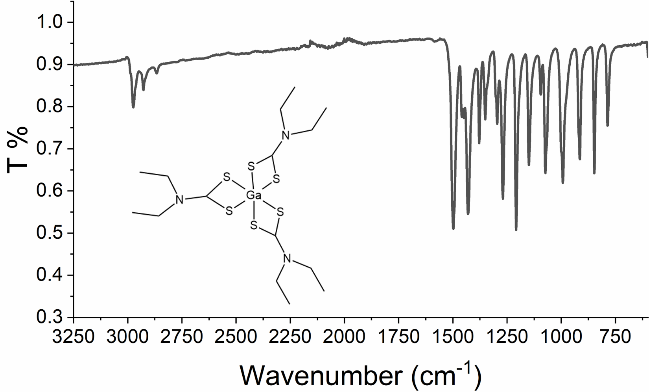

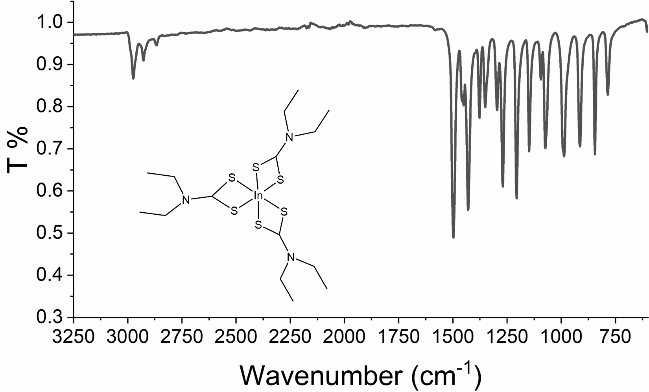


**Figure S3** - IR data of all synthesised metal diethyldithiocarbmate precursors used in the synthesis of HE metal sulfides.

# Thermogravimetric analysis and structure of the utilised precursors


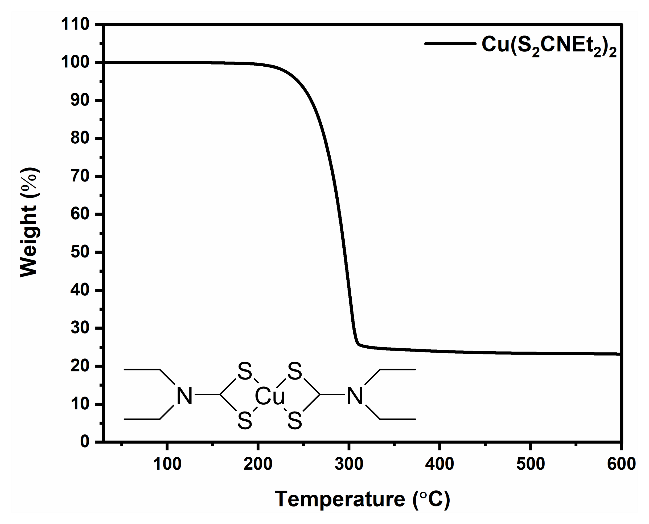

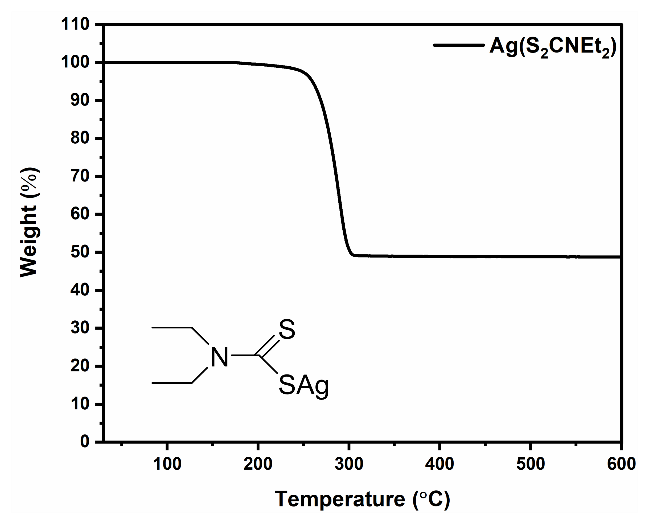

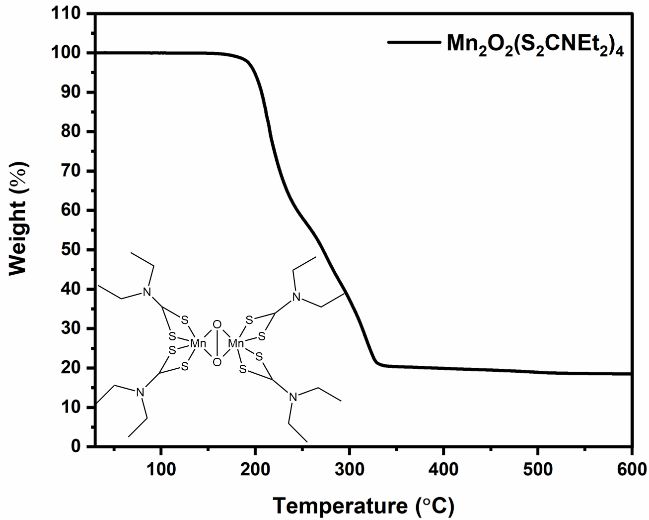

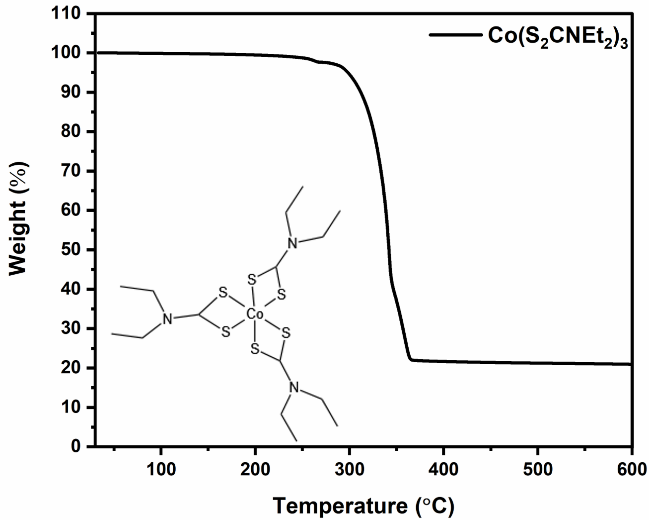

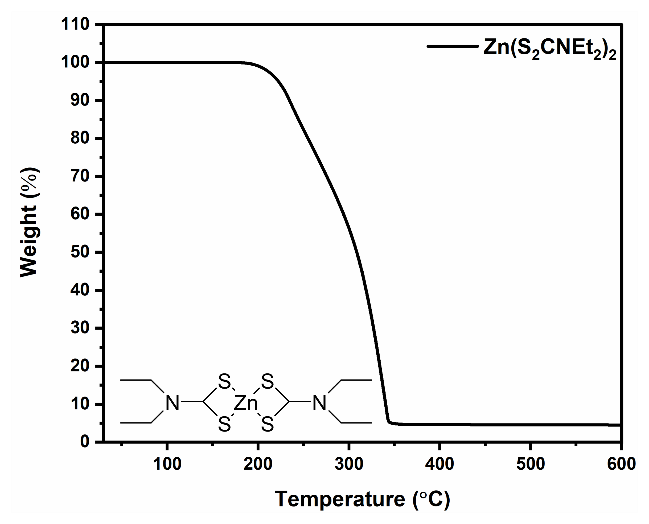

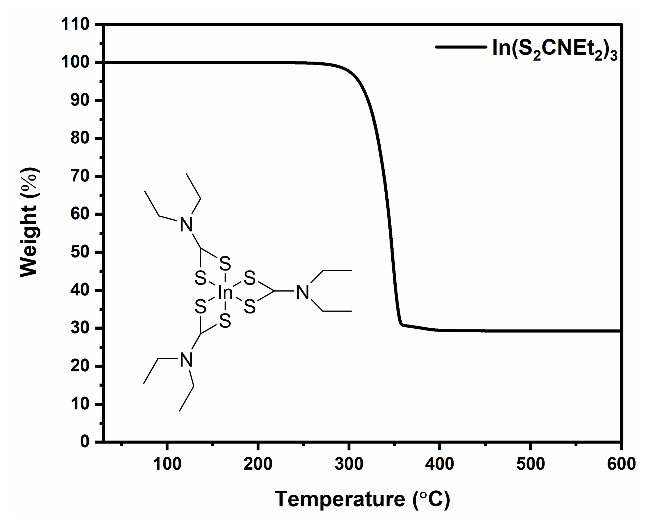


**(f)**

**(e)**

**(d)**

**(c)**

**(b)**

**(a)**


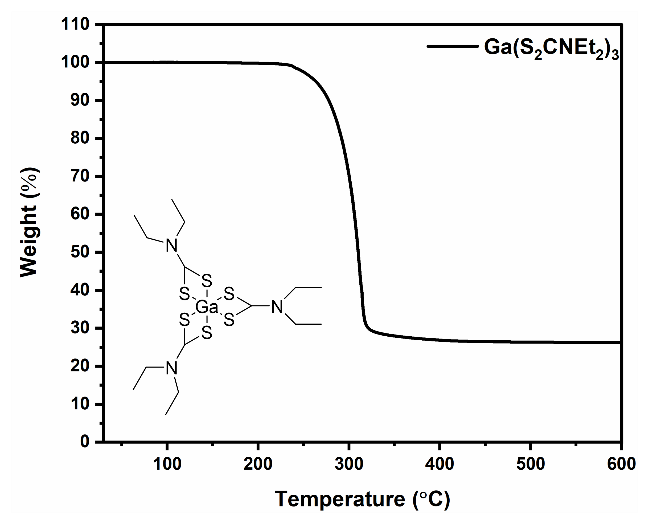




**(h)**

**(g)**

**Figure S4** - TGA curves of all metal dithocarbamate precursors where (a) Cu(DTC)_2_, (b) Ag(DTC), (c) Mn(DTC)_2_, (d) Co(DTC)_2_, Zn(DTC)_2_, In(DTC)_3_, Ga(DTC)_3_ are all shown. The structures of each precursor are also shown. All precursors have similar decomposition temperature.

# pXRD analysis of temperature for synthesis of HE materials

We note that the pXRD patterns are broad, it is likely this is due to the Scherrer broadening effect due to the small size of the particles, as shown in Figure S5 – 7.^11^

**(a)**







**(b)**

**Figure S5** – Figure showing the pXRD patterns of HE (a) (CuZnInGa)S and (b) (CuZnCoInGa)S powders synthesised at temperatures of 450, 500 and 550 °C.

# pXRD analysis of time for synthesis of HE materials





**Figure S6** – Figure showing the effect on decomposition time for the (AgCuInGa)S over a 1-hour and 5-hour period of time.

# p-XRD of stoichiometric Ag addition

Impurities of elemental Ag have been previously reported with the Ag diethydithiocarbamate precursor, in the presence of oleyamine as a surfactant.^12^ Similar low atomic solubility of Pb(S/Te) was reported in Pb(S/Se/Te) material, due to the high internal strain energy ($\Delta H_{S}$).^13^ Here, it is expected that the presence of elemental Ag indicates that the generated Ag_2_S further decomposes. This is expected beyond the solubility limit for Ag_2_S within the multi-metal sulfide. The poor solubility (low $S_{mix}$) of the Ag_2_S within these HES systems is likely due to the large size of the Ag^+^ cation (1.14 Å), which is larger than In^3+^ (0.8 Å), and significantly larger than Cu^+^ (0.60 Å), Zn^2+^ (0.60 Å), and Ga^3+^ (0.62 Å­) cations.^14^ This result, determining that a ‘size-compatibility’ of the Ag^+^ is present, has significant and wider implications for all multi-metal chalcogenides, and potentially multi-metal oxides, not just high-entropy metal sulfides. A molar ratio of 0.25 was therefore selected in the four and five metal sulfide material synthesis. A molar ratio of 0.5 was selected for the seven-metal sulfide synthesis as the extra elements was expected to yield a greater configurational entropy to further drive solubility of the large Ag^+^ cations through the sulfide lattice. This was also observed as the pXRD pattern of the seven metal found no presence of Ag metal (Figure 2(c)).











**Figure S7** – PXRD patterns of 5-metal HES (AgCuZnInGa)S with different silver ratio: 1, 0.75, 0.5, 0.25. Containing a major sphalerite phase and elemental silver contamination. Elemental silver content reduced by adding less silver dithiocarbamate precursor.





**Figure S8**. pXRD pattern of the 7-metal (AgCoMnCuZnInGa)S synthesised at 500 °C and 1000 °C for 1 hour.

# Rietveld refinements of pXRD patterns















**Figure S9**. Rietveld refinements of (a) (AgCuInGa)S, (b) (AgZnInGa)S, (c) (CuZnInGa)S, (d) (AgCuZnInGa)S, (e) (CoCuZnInGa)S, (f) (CuMnZnInGa)S.





**Figure S10**. Rietveld refinement of (AgCuZnCoMnInGa)S.

**Table S2**. Lattice Parameters of Rietveld Refinement to Sphalerite Structure.

| **Sphalerite Fitting** | **a** | **b** | **c** | **%** | **RWP** | **GOF** |
| --- | --- | --- | --- | --- | --- | --- |
| AgCuInGaS | 5.34 | 5.34 | 5.34 | 19.5 | 3.08 | 1.73 |
| AgZnInGaS | / | / | / | / | / | / |
| CuZnInGaS | / | / | / | / | / | / |
| AgCuZnInGaS | / | / | / | / | / | / |
| CuCoZnInGaS | 5.4 | 5.4 | 5.4 | 85.6 | 2.25 | 1.55 |
| CuMnZnInGaS | 5.46 | 5.46 | 5.46 | 86.7 | 3.46 | 2.61 |
| AgCoMnCuZnInGaS | / | / | / | / | / | / |
| α = β = γ = 90° | | | | | | |

**Table S3**. Lattice Parameters of Rietveld Refinement to Wurtzite Structure.

| **Wurtzite Fitting** | **a** | **b** | **c** | **%** | **RWP** | **GOF** |
| --- | --- | --- | --- | --- | --- | --- |
| AgCuInGaS | / | / | / | / | / | / |
| AgZnInGaS | 3.83 | 3.83 | 6.06 | 23.4 | 3.29 | 1.86 |
| CuZnInGaS | 3.81 | 3.81 | 6.03 | 13.5 | 2.68 | 1.58 |
| AgCuZnInGaS | 3.77 | 3.77 | 7.05 | 9.8 | 3.95 | 2.74 |
| CuCoZnInGaS | 3.81 | 3.81 | 6.22 | 4.2 | 2.25 | 1.55 |
| CuMnZnInGaS | 3.88 | 3.88 | 6.38 | 6.1 | 3.46 | 2.61 |
| AgCoMnCuZnInGaS | / | / | / | / | / | / |
| α = β = 90°, γ = 120° | | | | | | |

**Table S4**. Lattice Parameters of Rietveld Refinement to Chalcopyrite Structure.

| **Chalcopyrite Fitting** | **a** | **b** | **c** | **%** | **RWP** | **GOF** |
| --- | --- | --- | --- | --- | --- | --- |
| AgCuInGaS | 5.4 | 5.4 | 11 | 80.5 | 3.08 | 1.73 |
| AgZnInGaS | 5.44 | 5.44 | 10.99 | 76.6 | 3.29 | 1.86 |
| CuZnInGaS | 5.39 | 5.39 | 11.1 | 86.5 | 2.68 | 1.58 |
| AgCuZnInGaS | 5.38 | 5.38 | 11.02 | 90.2 | 3.95 | 2.74 |
| CuCoZnInGaS | 5.33 | 5.33 | 11.05 | 10.2 | 2.25 | 1.55 |
| CuMnZnInGaS | 5.49 | 5.49 | 11.03 | 7.2 | 3.46 | 2.61 |
| AgCoMnCuZnInGaS | 5.48 | 5.48 | 11.03 | 100% | 4.03 | 2.62 |
| α = β = γ = 90° | | | | | | |

# SEM-EDX maps of four and five metal sulfide HE material

## (CuZnInGa)S


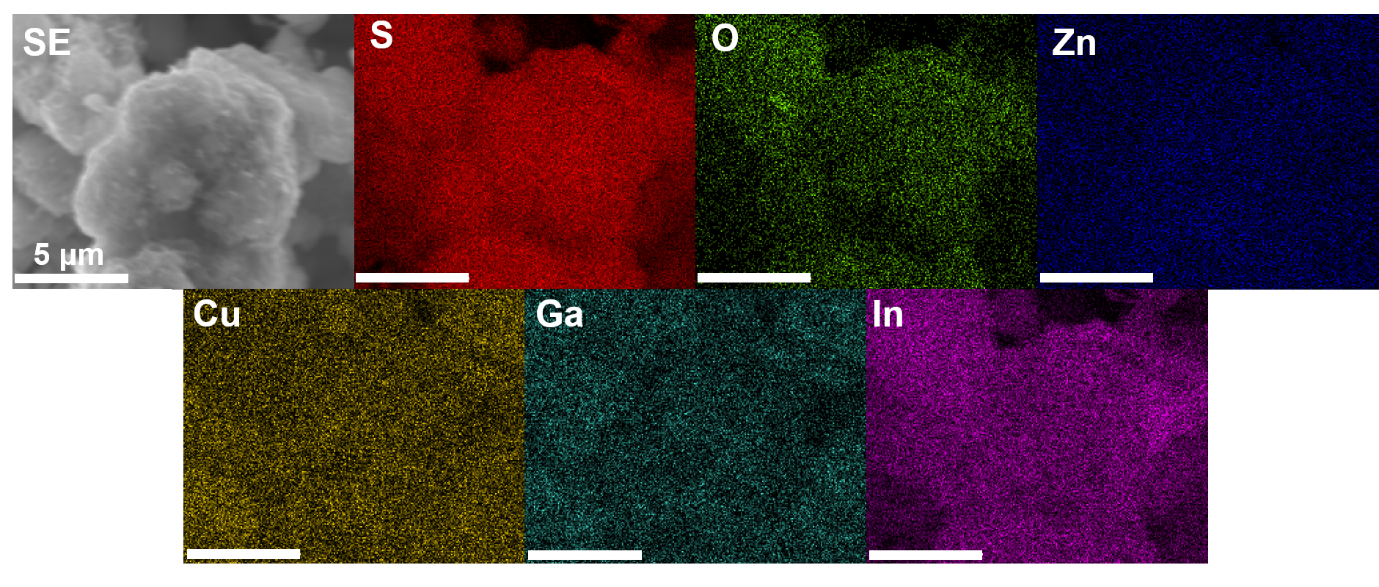


**Figure S11** – SEM image and EDX maps (20kV) of (CuZnInGa)S powders.

## (AgZnInGa)S


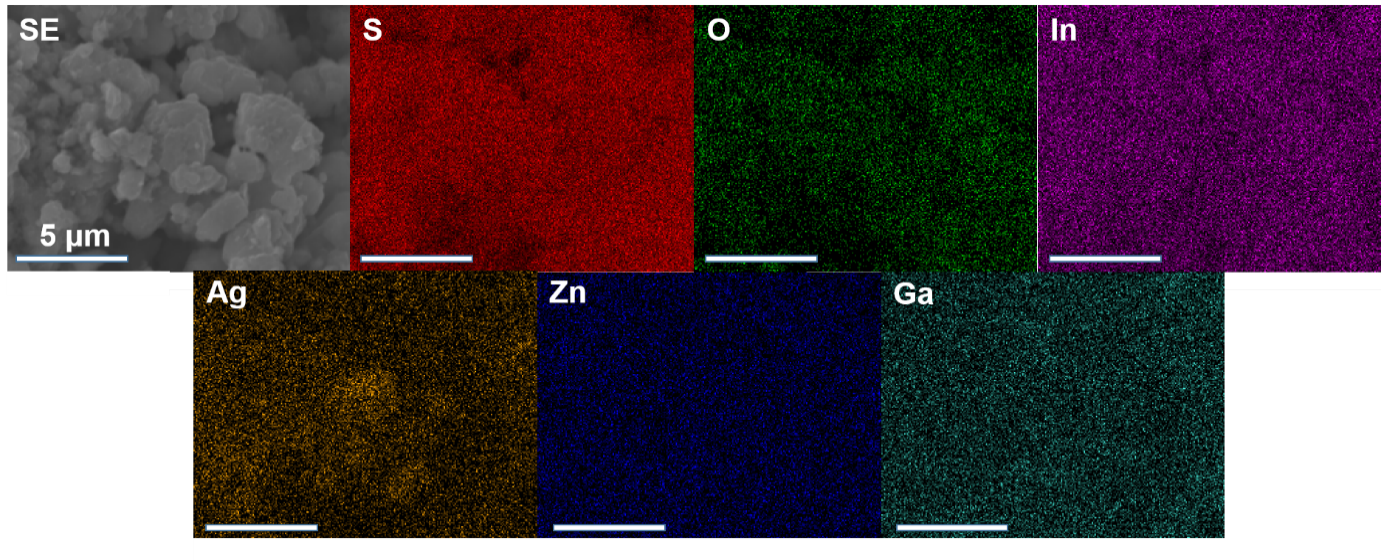


**Figure S12** – SEM image and EDX maps (20kV) of (AgZnInGa)S powders.

## (CuAgInGa)S


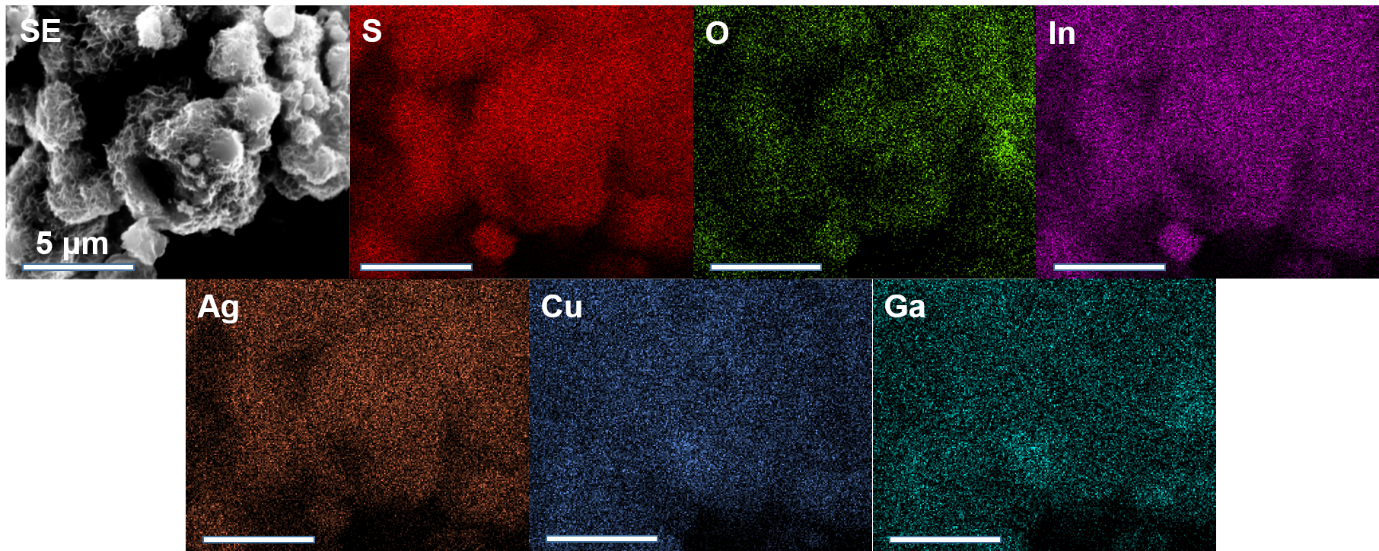


**Figure S13** – SEM image and EDX maps (20kV) of (CuAgInGa)S powders.

## (CuMnZnInGa)S


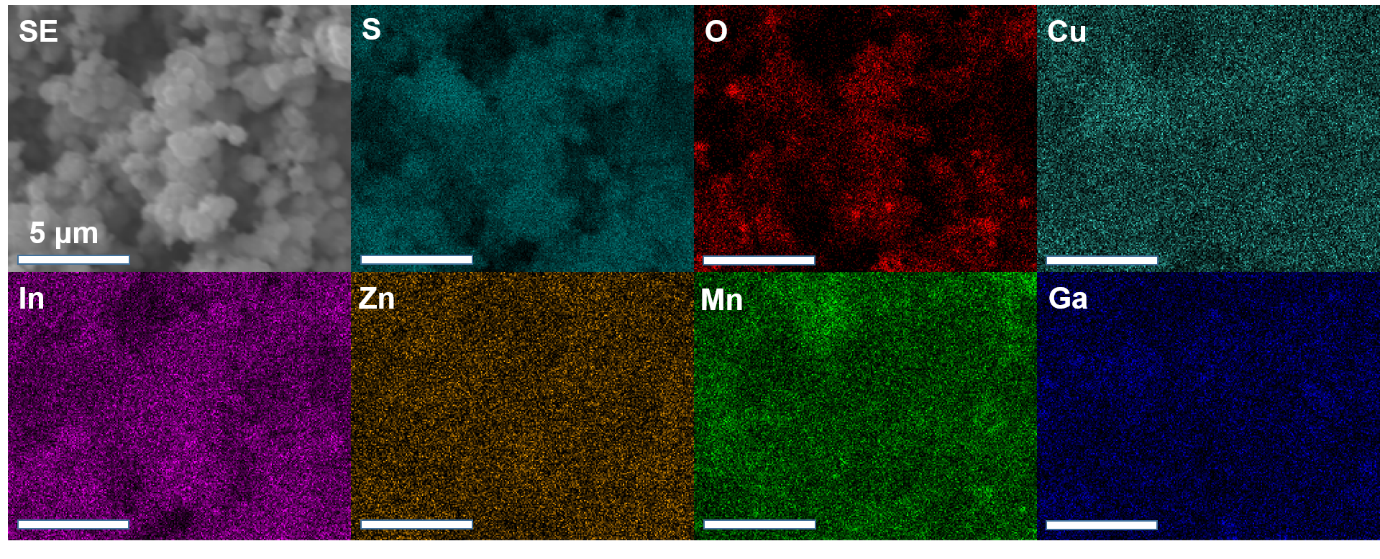


**Figure S14** – SEM image and EDX maps (20kV) of (CuMnZnInGa)S powders.

## (CuAgZnInGa)S


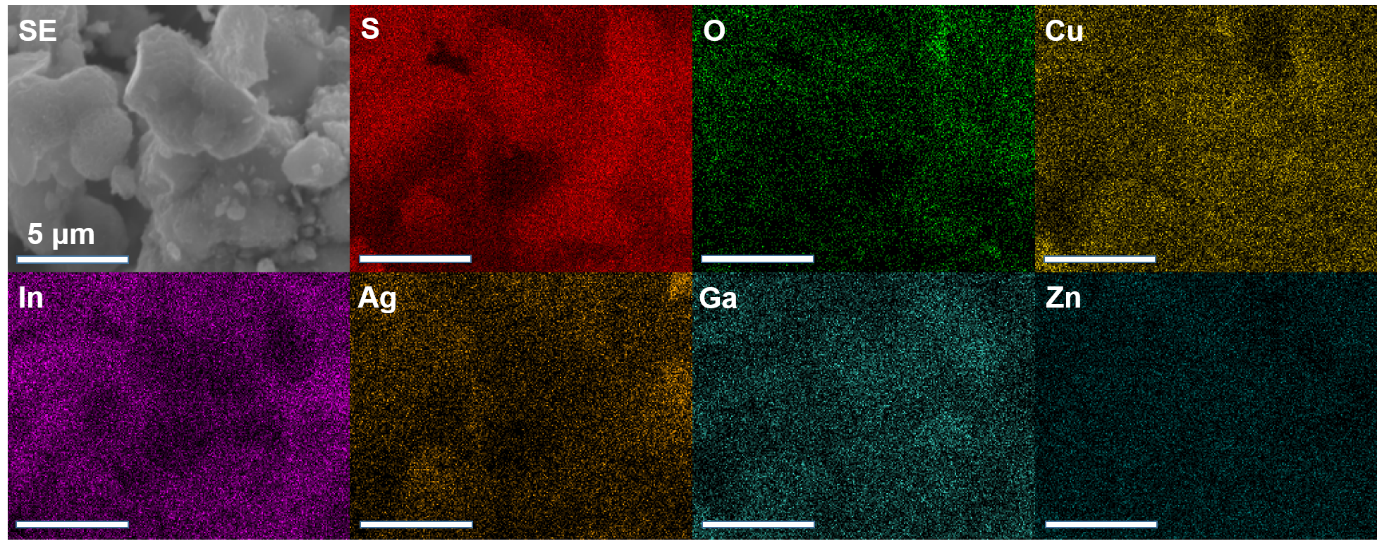


**Figure S15** – SEM image and EDX maps (20kV) of (CuAgZnInGa)S powders.

## (CuCoZnInGa)S


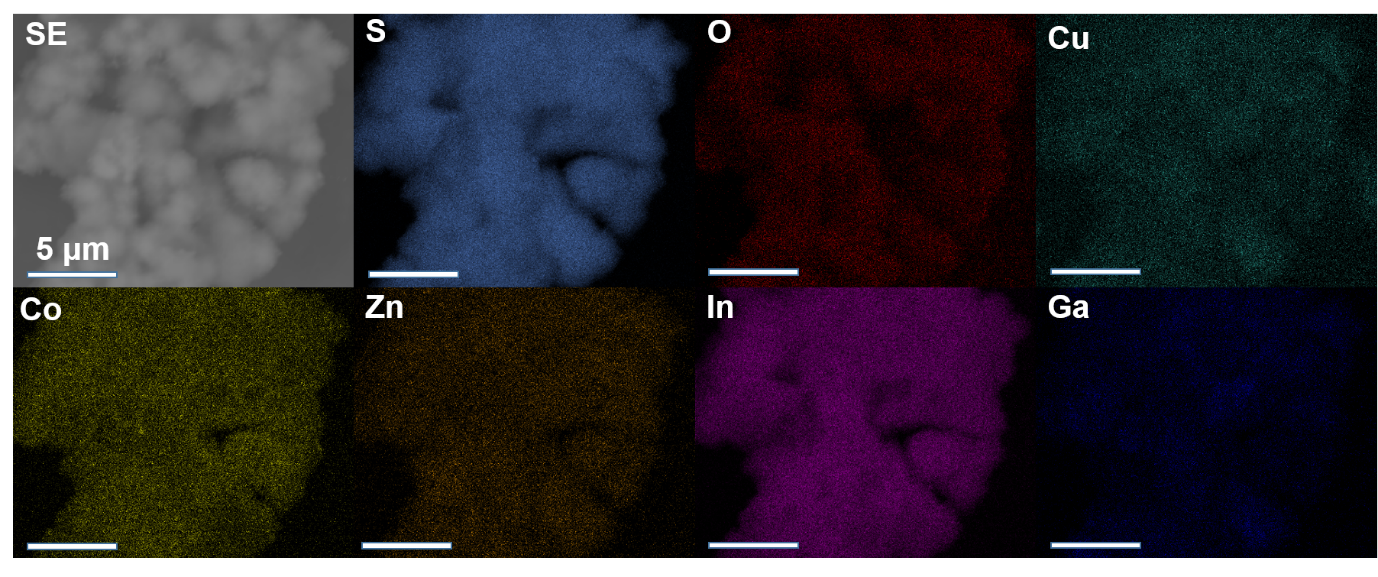


**Figure S16** – SEM image and EDX maps (20kV) of (CuCoZnInGa)S powders.

# STEM-EDX maps of four and five metal sulfide HE material

## (CuZnGaIn)S


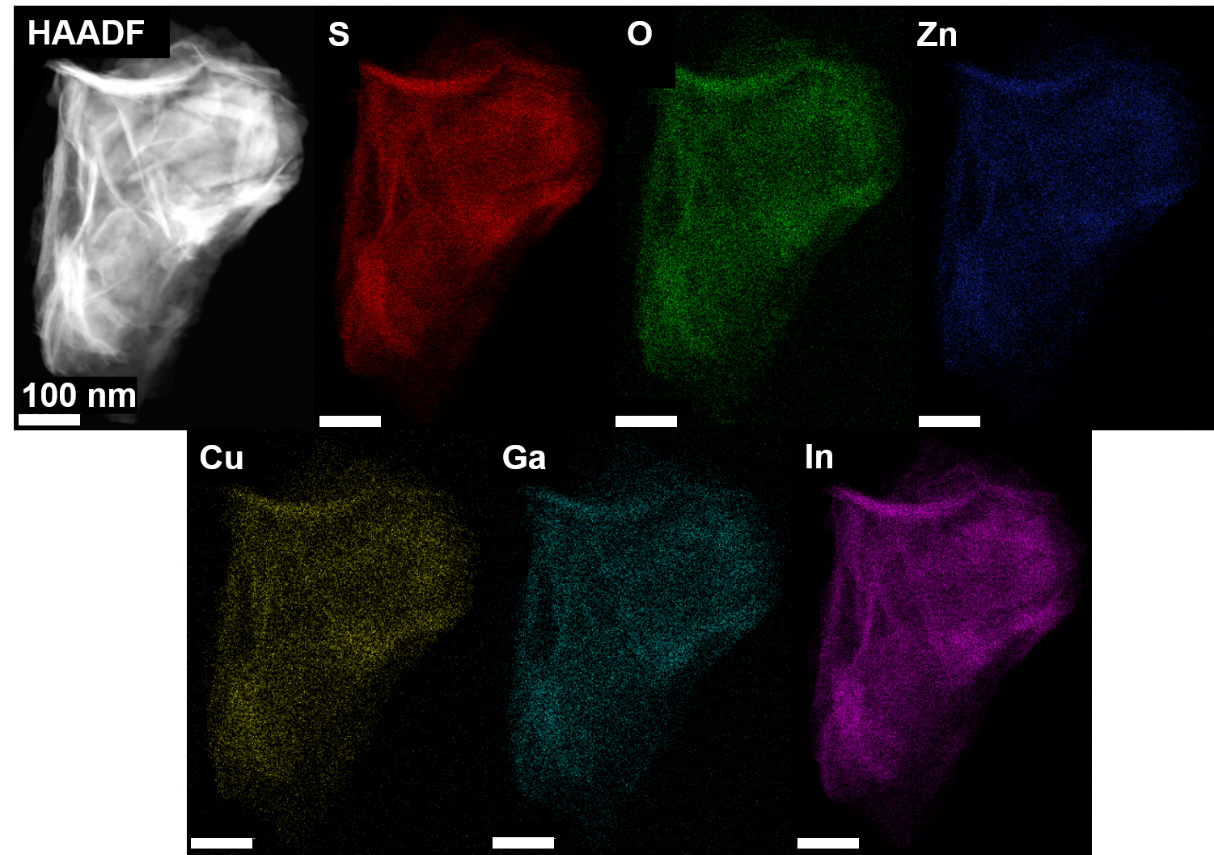


**Figure S17** – TEM image and EDX maps (200kV) of (CuZnGaIn)S powders.


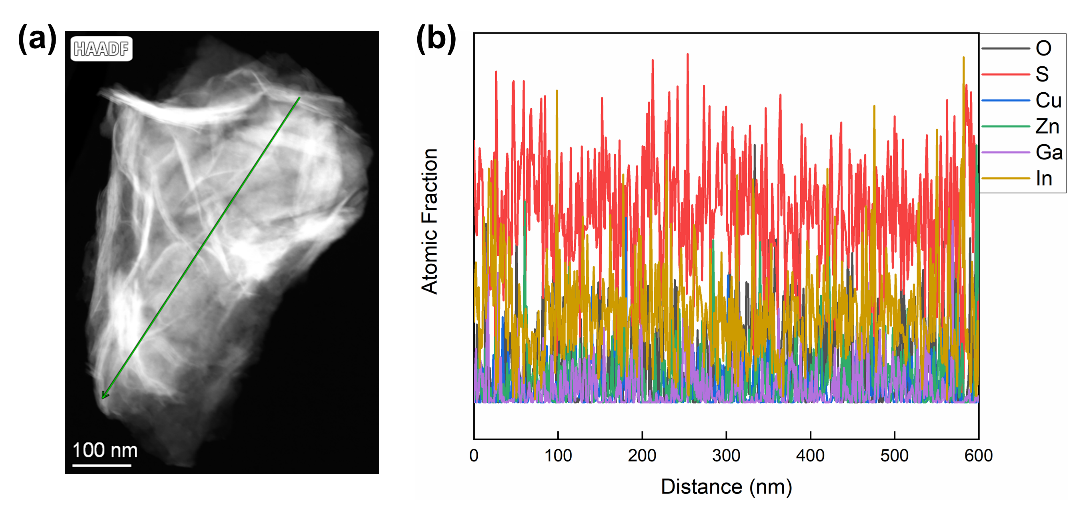


HAADF

**Figure S18**. (a)HAADF image and (b) EDX line scan of (CuZnGaIn)S particle.

## (AgZnInGa)S


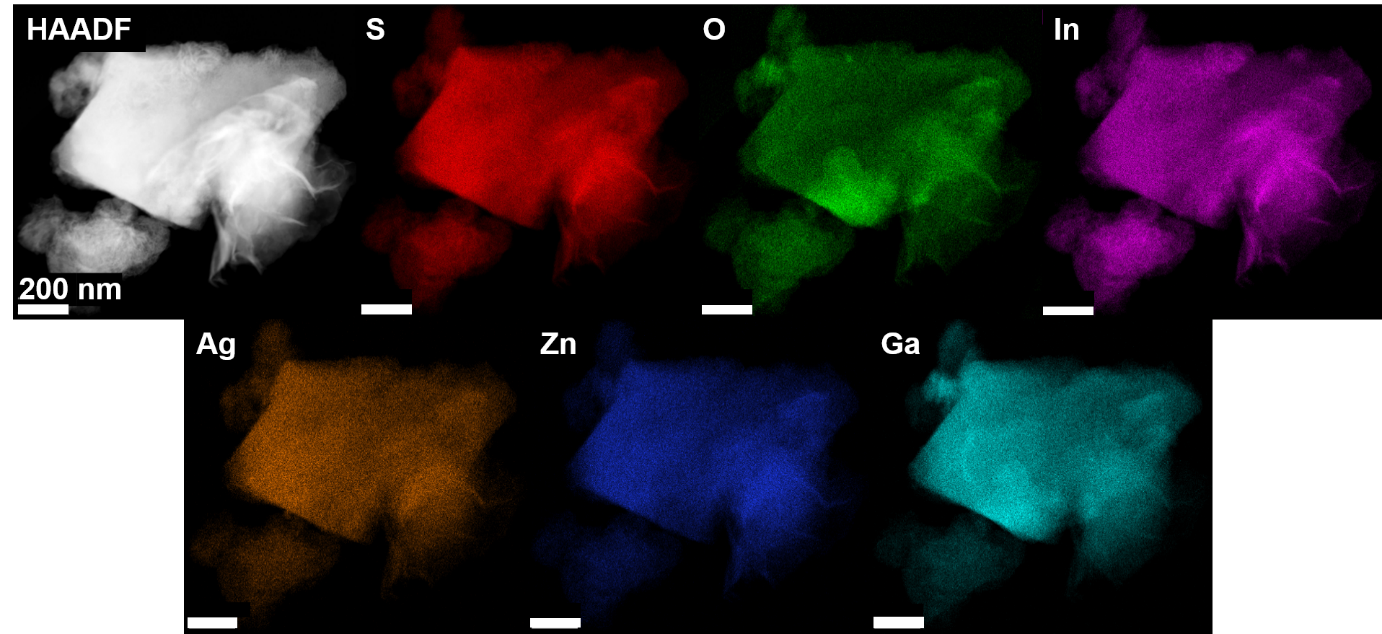


**Figure S19** - TEM image and EDX maps (200kV) of (AgZnInGa)S powders.


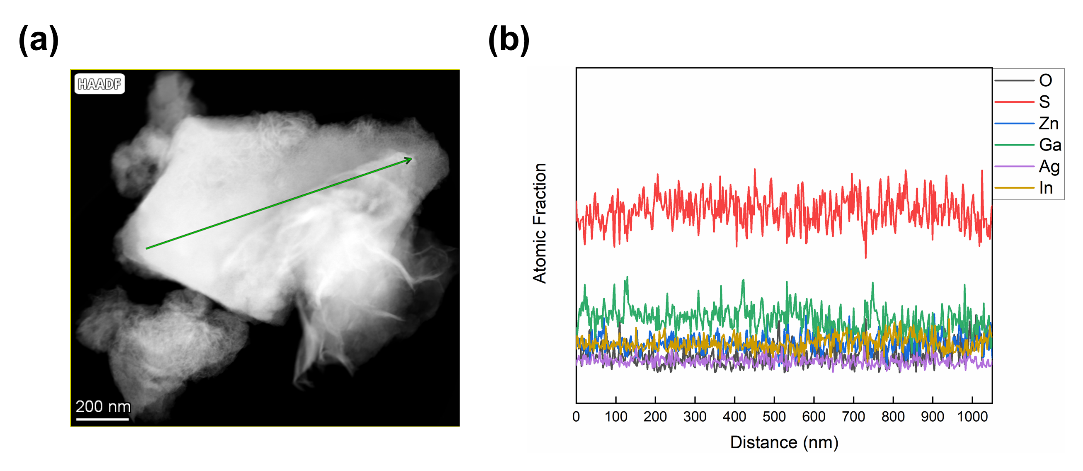


HAADF

**Figure S20**. (a)HAADF image and (b) EDX line scan of (AgZnInGa)S particle.

## (CuAgInGa)S


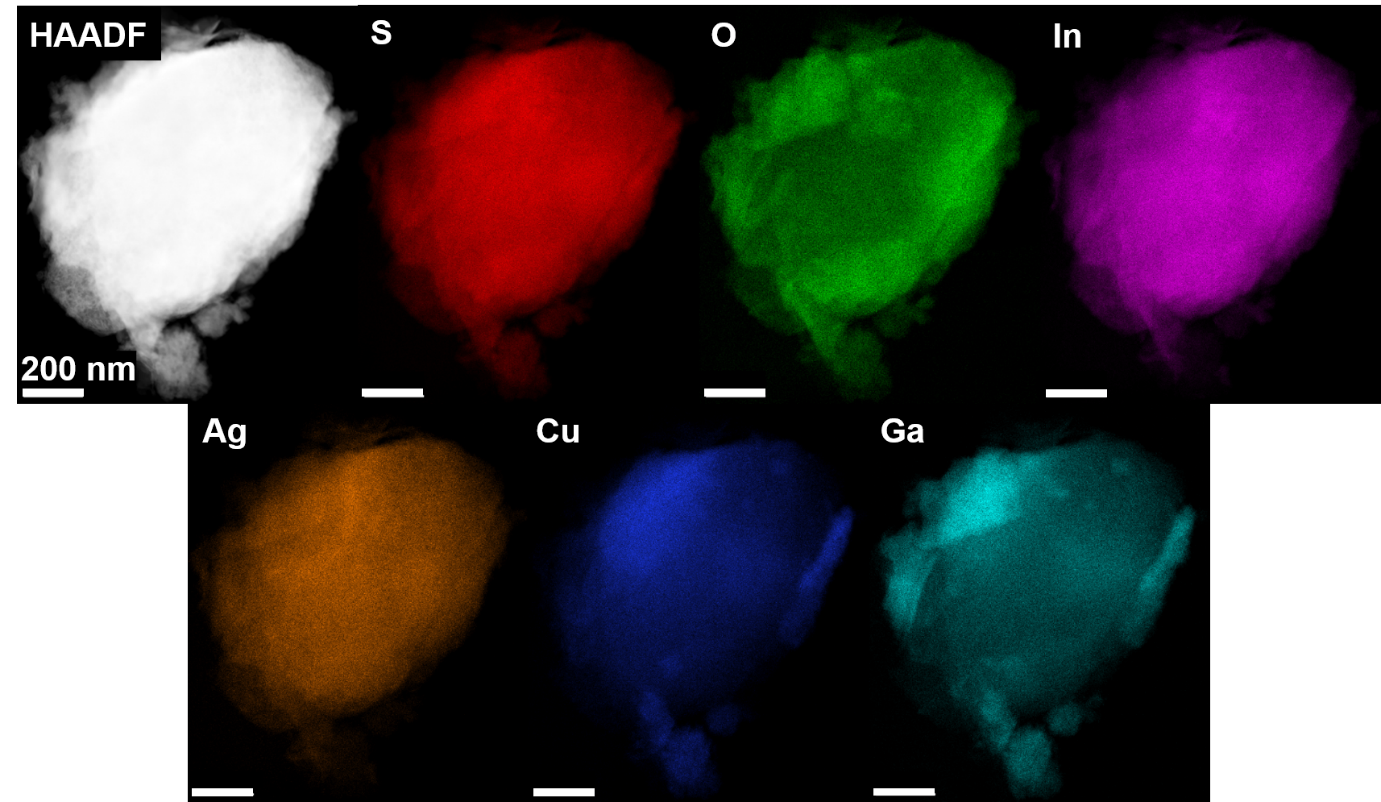


**Figure S21** - TEM image and EDX maps (200kV) of (CuAgInGa)S powders.


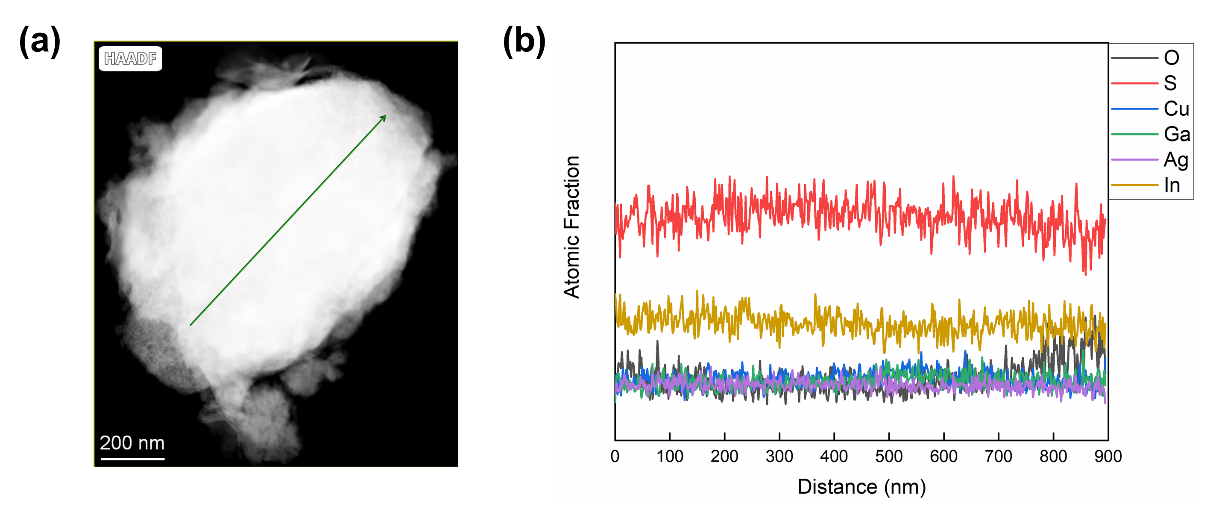


HAADF

**Figure S22**. (a)HAADF image and (b) EDX line scan of (CuAgInGa)S particle.

## (CuMnZnInGa)S

STEM-EDX analysis of the (CuMnZnInGa)S system found some possible Mn and O localisation and segregation from the other elements. This was not detected in the SEM-EDX analysis and demonstrates the requirement for this analysis at multiple length scales. It is likely that the species present is MnSO_4_ due to surface oxidation, as MnSO_4_ was detected by XPS of our 7-metal entropy stabilized system.


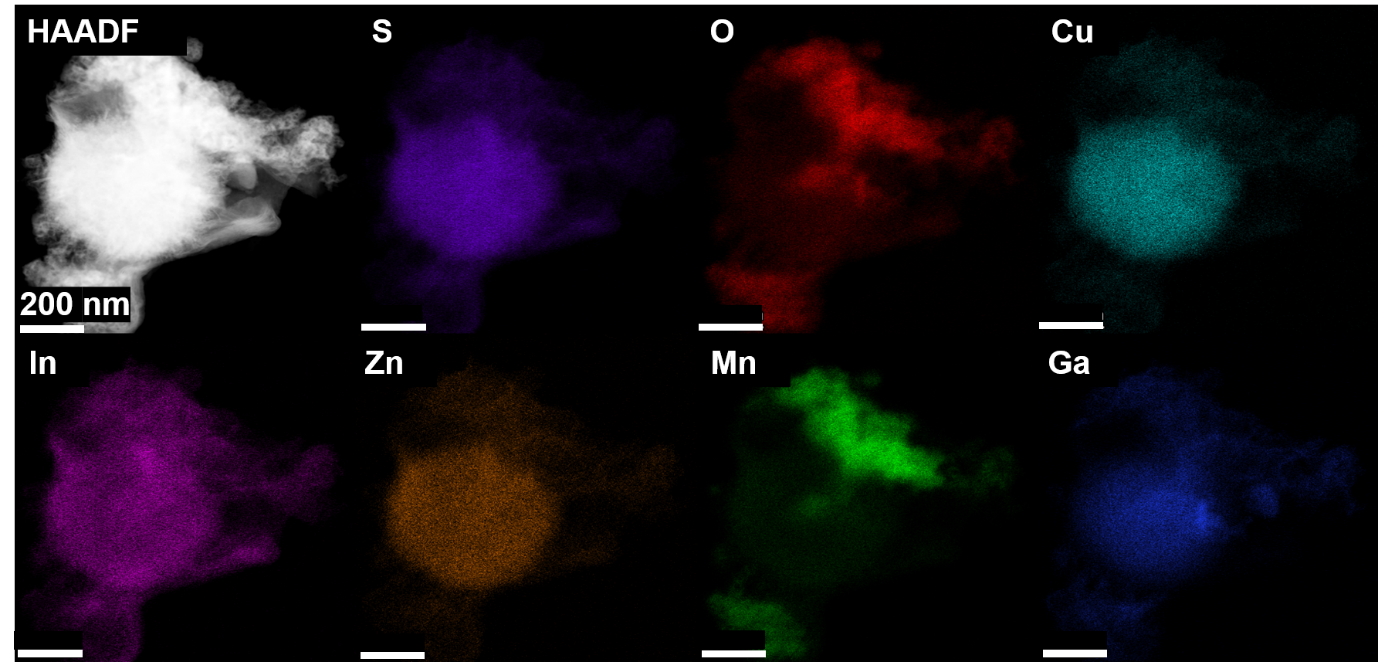


**Figure S23** - TEM image and EDX maps (200kV) of (CuMnZnInGa)S powders.


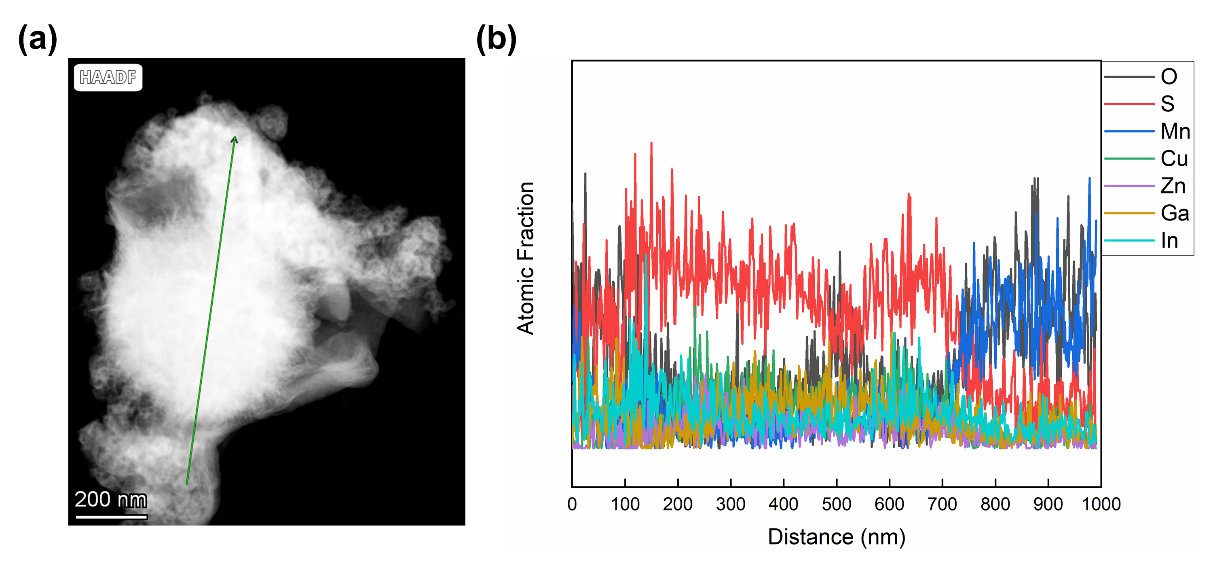


HAADF

**Figure S24**. (a)HAADF image and (b)EDX line scan of (CuMnZnInGa)S particle.

## (CuAgZnInGa)S


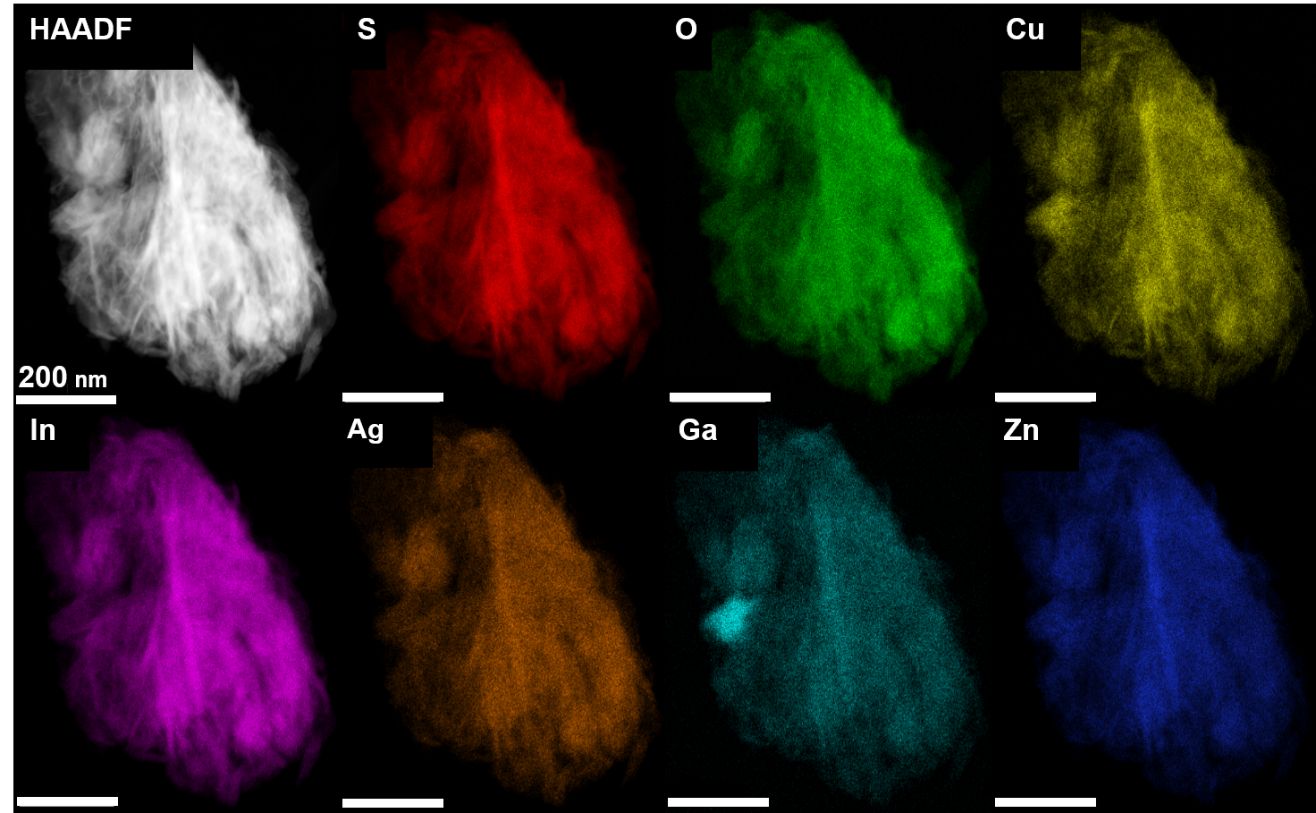


**Figure S25** - TEM image and EDX maps (200kV) of (CuAgZnInGa)S powders.


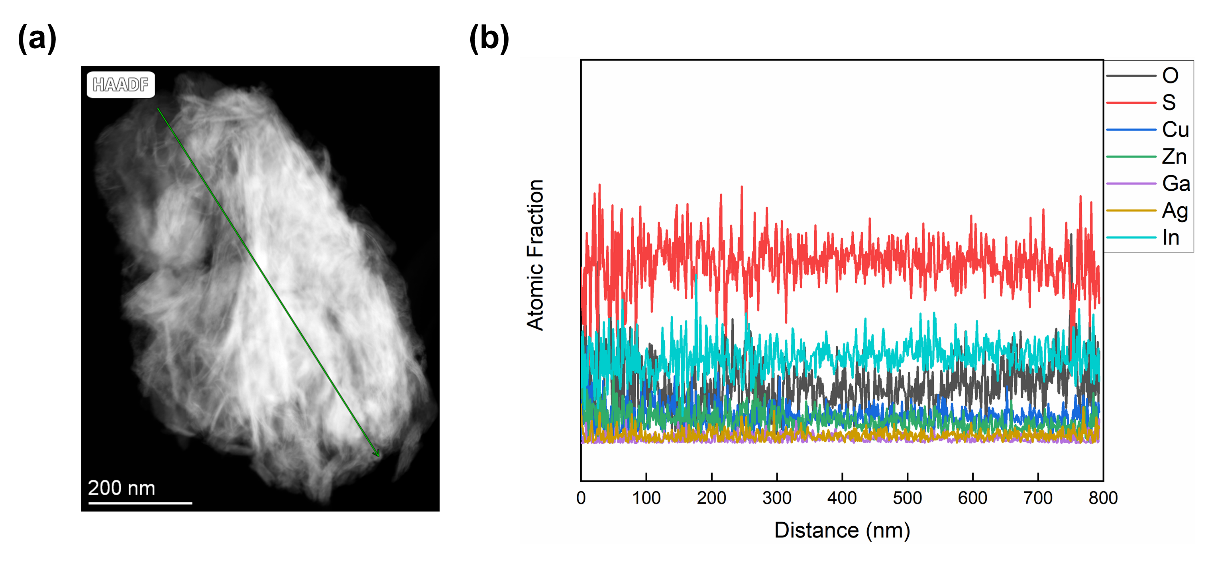


HAADF

**Figure S26**. (a)HAADF image and (b) EDX line scan of (CuAgZnInGa)S particle.

## (CuCoZnInGa)S


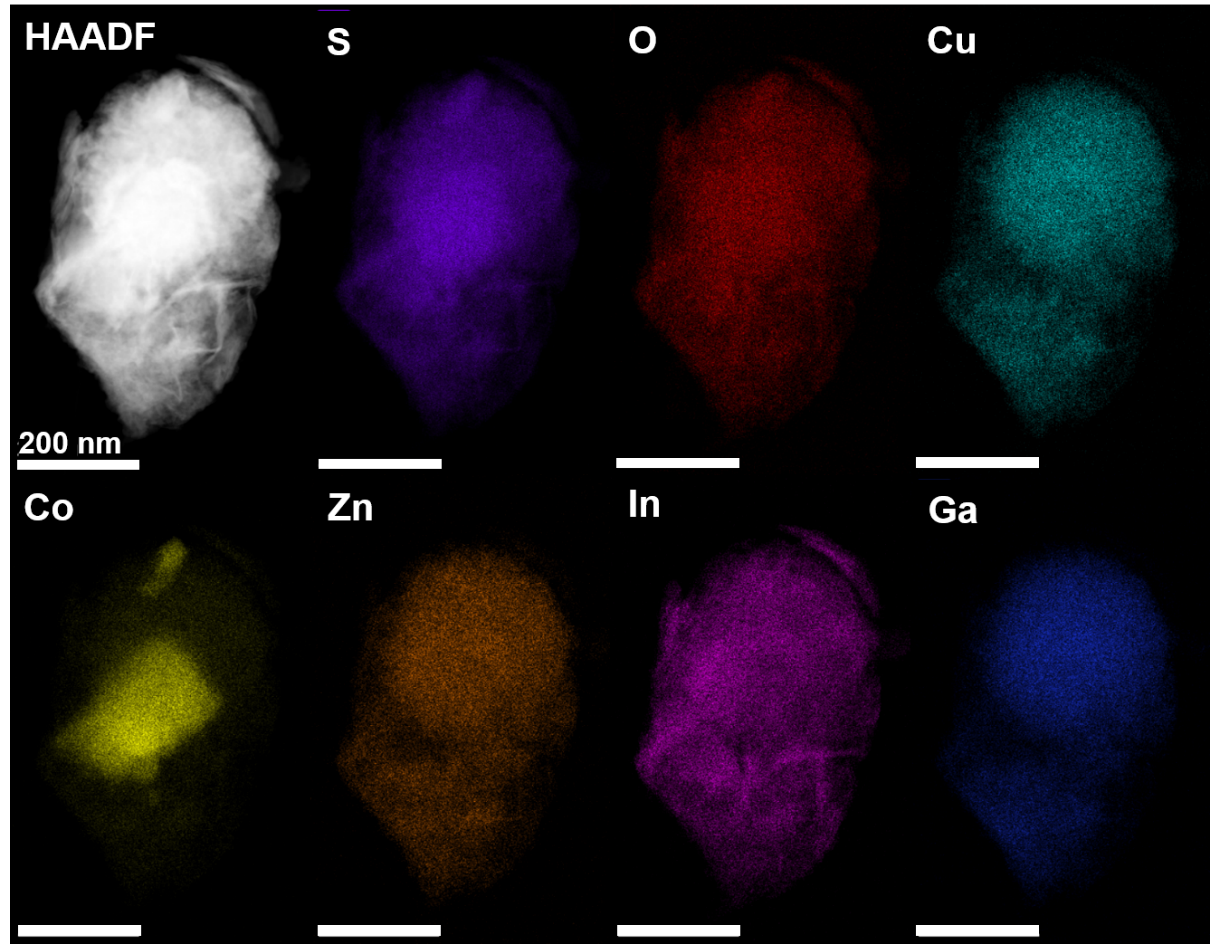


**Figure S27** – TEM image and EDX maps (200kV) of (CuCoZnInGa)S powders.


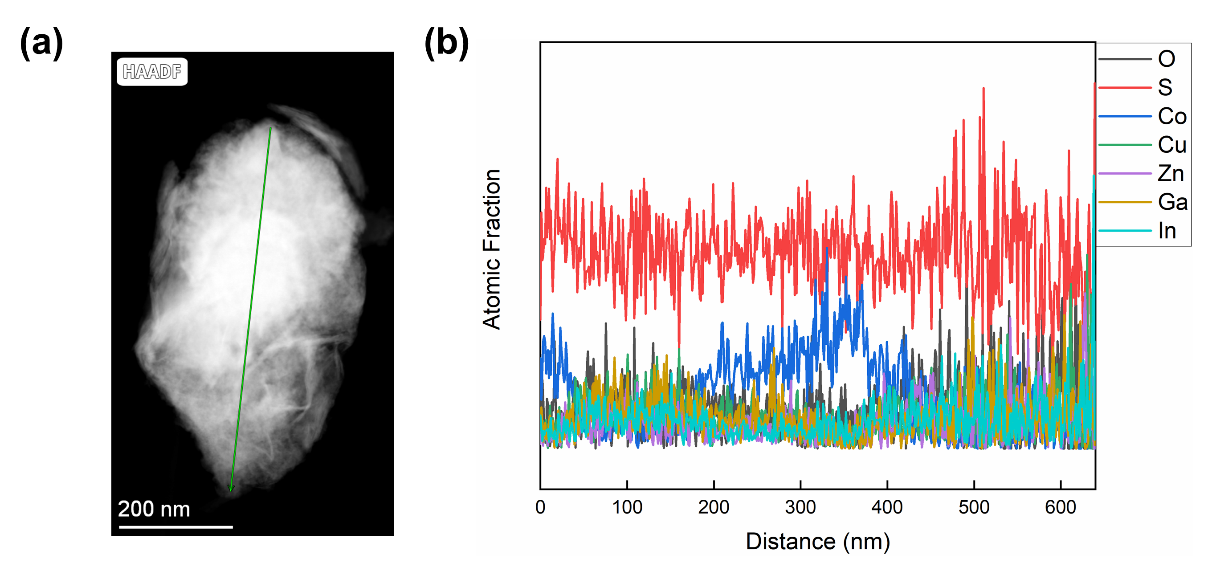


HAADF

**Figure S28**. (a)HAADF image and (b)EDX line scan of (CuCoZnInGa)S particle.


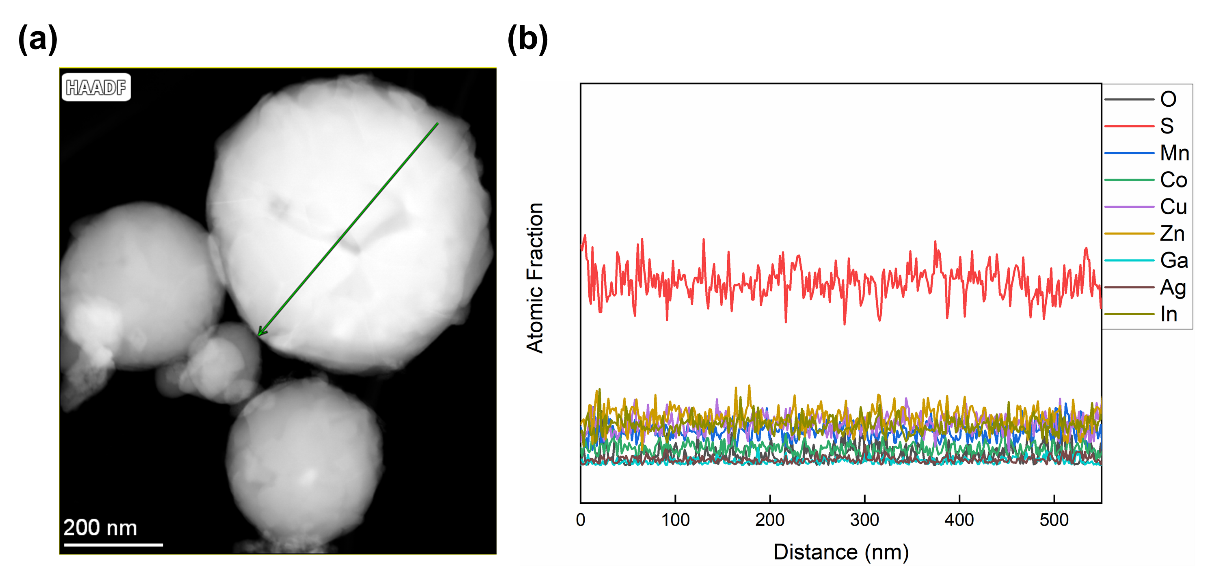


HAADF

**Figure S29**. (a)HAADF image and (b) EDX line scan of (AgCuZnCoMnInGa)S particle.

# Table of data for Microscale *vs* Nanoscale elemental composition

There are multiple reasons causing a difference in elemental ratio analysed by these 3 techniques. In SEM-EDX, an elemental concentration is calculated directly from sample to standard intensity ratios determined by least squares fitting of background filtered (removed) standard profiles to the sample spectrum. Followed by an XPP matrix correction (a Phi-Rho-Z approach) where the correction factors are dependent on the composition of the sample. The true concentrations have to be derived using an iterative procedure. The determined concentrations are used to calculate correction factors which are then used to derive more precise ‘estimates’ for the concentrations and so on until after successive iterations, a self-consistent set of concentrations and correction factors are obtained.

STEM-EDX quantification has been performed using the Velox software with a multi-polynomial model and parabolic background correction as well as Brown-Powell approximation for the ionization cross-section model.

Another reason could be a local difference in the materials. In STEM, the beam picked up one isolated small particle of *ca.* 500 nm diameter. Two particles at this scale can have different elemental ratio. While the SEM select an area of about 10 $\times$ 10 μm, which gives an average result.

XPS, which is very different from EDX, is a surface sensitive technique which only measures the surface to a depth of *ca.* 6 nm.^15^ The small measurement depth can result in a composition difference from EDX.

**Table S5 –** Normalised atomic percent of (CuAgMnCoZnInGa)S corresponding to the EDX maps of SEM and STEM in Figure 3.

| **Element** | **SEM-EDX** | **STEM-EDX** | **XPS (minus C and O)** |
| --- | --- | --- | --- |
| Cu | 10.6 ± 0.7 | 11.2 ± 2.2 | 4.88 ± 0.62 |
| Ag | 4.6 ± 0.4 | 0.7 ± 0.1 | 16.77 ± 0.35 |
| Mn | 3.4 ± 0.6 | 8.6 ± 1.7 | 3.02 ± 0.44 |
| Co | 8.2 ± 0.6 | 5.0 ± 0.9 | 2.84 ± 0.62 |
| Zn | 10.3 ± 0.1 | 12.3 ± 2.3 | 3.91 ±0.35 |
| In | 10.8 ± 0.7 | 10.6 ± 1.8 | 13.75 ± 0.35 |
| Ga | 5.5 ± 1.7 | 0.9 ± 0.2 | 5.94 ± 0.27 |
| S | 46.6 ± 7.2 | 50.7 ± 11.7 | 48.89 ± 2.93 |

# XPS


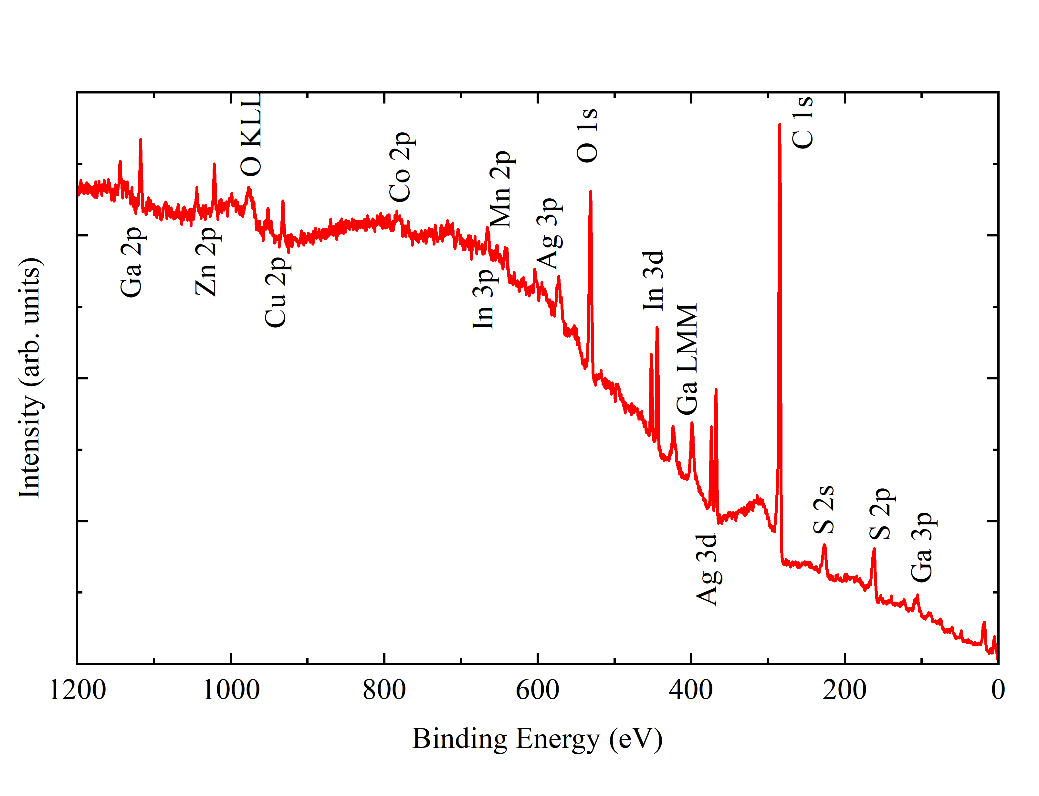


**Figure S30** – XPS Survey spectrum with the strongest photoelectron peaks labelled.

**Table S6** - Excluding C and O, the total percentage of metals and the metal to sulfur ratio.

|  | Metal % | Metal:S |
| --- | --- | --- |
| Position 1 | 6.62 | 1.08 |
| Position 2 | 4.91 | 1.01 |
| Average | **5.77** | **1.04** |

# Table of data for configurational entropy calculation

**Table S7** – Table of data relating to Figure 4 in the main text.

| Metal sulfide system | 2-metal | 3-metal | 4-metal | 5-metal | 6-metal | 7-metal |
| --- | --- | --- | --- | --- | --- | --- |
| Expected Entropy / R | 0.35 | 0.52 | 0.69 | 0.81 | 0.89 | 0.97 |
| (AgZnInGa)S | - | - | 0.61 | - | - | - |
| (CuAgInGa)S | - | - | 0.67 | - | - | - |
| (CuZnInGa)S | - | - | 0.69 | - | - | - |
| (CuAgZnInGa)S | - | - | - | 0.73 | - | - |
| (CuCoZnInGa)S | - | - | - | 0.79 | - | - |
| (CuZnMnInGa)S | - | - | - | 0.80 | - | - |
| (CuAgZnMnCoInGa)S | - | - | - | - | - | 0.93 |

# pXRD of three-element metal sulfides





**Figure S31**. p-XRD Patterns for 3-metal systems.





**Figure 32**. p-XRD pattern for (Cu_2_InGa)S.

# Electrochemical Impedance analysis





**Figure S33**. Electrochemical Impedance Spectroscopy Nyquist plots of investigated carbon-black enhanced metal sulfides. Inset is the fitting model used.

# C_dl_ analysis


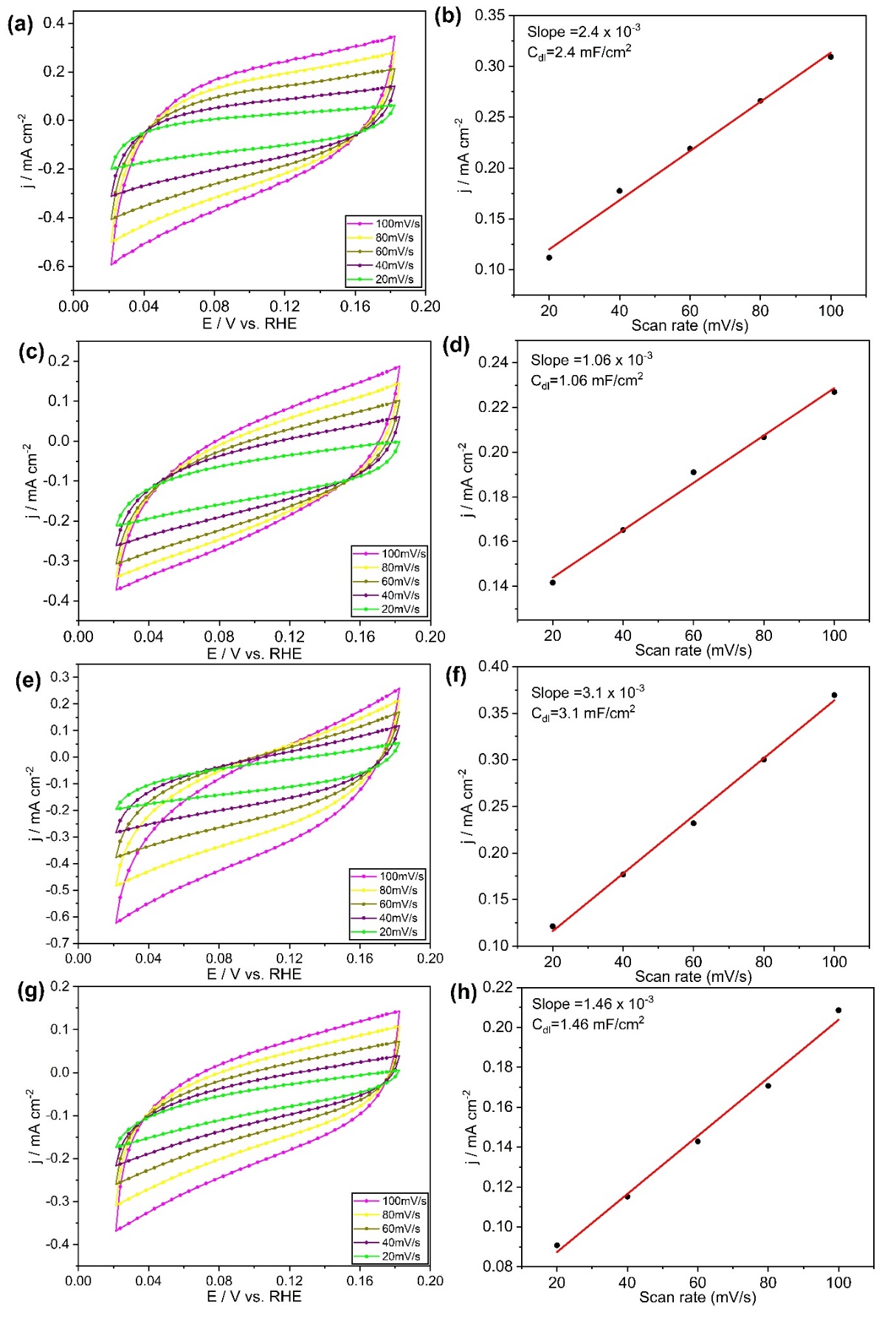


**Figure S34**. (a, c, e, g) CV plots of (AgCuZnMnCoInGa)S, (CoInGa)S, (CuInGa)S, and (ZnInGa)S, respectively, at scan rate 20, 40, 60, 80, and 100 mV s^−1^. (b, d, f, h) the corresponding estimated double-layer capacitor (C_dl_) of (AgCuZnMnCoInGa)S, (CoInGa)S, (CuInGa)S, and (ZnInGa)S.

# Table of data for electrocatalysis

**Table S8**. Table of data for electrocatalysis.

| **Material** | **Onset Overpotential / mV** | **η_10_ / mV** |
| --- | --- | --- |
| MnS | 396 | 535 |
| Ag2S | 382 | 529 |
| (CoInGa)S | 435 | 569 |
| (CuInGa)S | 562 | Not reached |
| (ZnInGa)S | 441 | Not reached |
| (AgCuZnMnCoInGa)S | 309 | 455 |
| MnS@20%CB | 305 | 470 |
| Ag_2_S@20%CB | 287 | 452 |
| (CoInGa)S@20%CB | 314 | 463 |
| (CuInGa)S@20%CB | 435 | 527 |
| (ZnInGa)S@20%CB | 491 | 608 |
| (AgCuZnMnCoInGa)S@20%CB | 68 | 248 |

# Table of data for literature electrocatalysis

**Table S9**. Comparisons between reported metal sulphides HER electrocatalysts.

| **System** | **η_10_ / mV** | **Electrolyte** | ***R*_ct_ / Ω** | **Reference** |
| --- | --- | --- | --- | --- |
| layered CuS@C (nanosheets) | 128 | 0.5 M H­_2_SO_4_ | 55 | ^16^ |
| Ag_2_S/CuS (nanoporous) | 193 | 0.5 M H­_2_SO4 | Not measured | ^17^ |
| Ag_2_S/Ag (micro region wire like) | 199 | 0.5 M H­_2_SO_4_ | 52.9 | ^18^ |
| Cu/Cu_2_O/Cu_2_S (nanotubes) | 86 | 0.5 M H­_2_SO_4_ | Not measured | ^19^ |
| CoS (nanosheet) | 198 | 0.5 M H_2_SO_4_ | 8.8 | ^20^ |
| CoS_2_  N doped CoS_2_ (Flims) | 124  97 | 0.5 M H_2_SO_4_ | 1.627  1.576 | ^21^ |
| CuCo_2_S_4_ (nanocluster) | 135 | 0.5 M H_2_SO_4_ | 71 | ^22^ |
| (CoInGa)S@20%CB | 464 | 0.5 M H_2_SO_4_ | 0.35 | Our work |
| (CuInGa)S@20%CB | 527 | 0.5 M H_2_SO_4_ | 0.61 | Our work |
| (ZnInGa)S@20%CB | 608 | 0.5 M H_2_SO_4_ | 0.67 | Our work |
| (AgCuZnMnCoInGa)S@20%CB | 248 | 0.5 M H_2_SO_4_ | 0.35 | Our work |

**Table S10**. Long-time comparisons between reported metal sulphides HER electrocatalysts.

| **System** | **Time/Test** | Overpotential / mV | Current density / mA cm^-2^ | **Reference** |
| --- | --- | --- | --- | --- |
| layered CuS@C (nanosheets) | 20 h | 128 | 10 | *^15^* |
| Ag_2_S/CuS (nanoporous) | 1000 cycle | ~ 193 | 10 | *^16^* |
| Ag_2_S/Ag (micro region wire like) | 1000 cycle | 199 | 10 | *^17^* |
| CoS (nanosheet) | 25 h | 198 | 10 | *^19^* |
| N doped CoS_2_ (Flims) | 50h | ~125  ~300 | 10  100 | *^20^* |
| CuCo_2_S_4_ (nanocluster) | 10h | 137 – 190 | 10 | *^21^* |

# References

1. Murtaza, G.; Alderhami, S.; Alharbi, Y. T.; Zulfiqar, U.; Hossin, M.; Alanazi, A. M.; Almanqur, L.; Onche, E. U.; Venkateswaran, S. P.; Lewis, D. J., Scalable and Universal Route for the Deposition of Binary, Ternary, and Quaternary Metal Sulfide Materials from Molecular Precursors. *ACS Applied Energy Materials* **2020,** *3* (2), 1952-1961.

2. Hendrickson, A. R.; Martin, R. L.; Rohde, N. M., Tris(dithiocarbamato) complexes of manganese(II), manganese(III), and manganese(IV). Electrochemical study. *Inorganic Chemistry* **1974,** *13* (8), 1933-1939.

3. Eagle, C. T.; Holder, G. N.; Goodman, A. B.; Hicks, P. E.; Shaber, K. P., Synthesis of bis(diethyldithiocarbamato)manganese(II) and tris(diethyldithiocarbamato)manganese(III). *The Chemical Educator* **2001,** *6* (3), 153-156.

4. Sarker, J. C.; Hogarth, G., Dithiocarbamate Complexes as Single Source Precursors to Nanoscale Binary, Ternary and Quaternary Metal Sulfides. *Chem Rev* **2021,** *121* (10), 6057-6123.

5. Qu, J.; Elgendy, A.; Cai, R.; Buckingham, M. A.; Papaderakis, A. A.; de Latour, H.; Hazeldine, K.; Whitehead, G. F. S.; Alam, F.; Smith, C. T.; Binks, D. J.; Walton, A.; Skelton, J. M.; Dryfe, R. A. W.; Haigh, S. J.; Lewis, D. J., A Low-Temperature Synthetic Route Toward a High-Entropy 2D Hexernary Transition Metal Dichalcogenide for Hydrogen Evolution Electrocatalysis. *Advanced Science* **2023,** *10* (14), 2204488.

6. Makin, F.; Alam, F.; Buckingham, M. A.; Lewis, D. J., Synthesis of ternary copper antimony sulfide via solventless thermolysis or aerosol assisted chemical vapour deposition using metal dithiocarbamates. *Scientific Reports* **2022,** *12* (1), 5627.

7. Higgins, E. P. C.; Papaderakis, A. A.; Byrne, C.; Cai, R.; Elgendy, A.; Haigh, S. J.; Walton, A. S.; Lewis, D. J.; Dryfe, R. A. W., High-Performance Nanostructured MoS2 Electrodes with Spontaneous Ultralow Gold Loading for Hydrogen Evolution. *The Journal of Physical Chemistry C* **2021,** *125* (38), 20940-20951.

8. Chen, R.; Yang, C.; Cai, W.; Wang, H.-Y.; Miao, J.; Zhang, L.; Chen, S.; Liu, B., Use of Platinum as the Counter Electrode to Study the Activity of Nonprecious Metal Catalysts for the Hydrogen Evolution Reaction. *ACS Energy Letters* **2017,** *2* (5), 1070-1075.

9. Elgendy, A.; Papaderakis, A. A.; Byrne, C.; Sun, Z.; Lauritsen, J. V.; Higgins, E. P. C.; Ejigu, A.; Cernik, R.; Walton, A. S.; Lewis, D. J.; Dryfe, R. A. W., Nanoscale Chevrel-Phase Mo6S8 Prepared by a Molecular Precursor Approach for Highly Efficient Electrocatalysis of the Hydrogen Evolution Reaction in Acidic Media. *ACS Applied Energy Materials* **2021,** *4* (11), 13015-13026.

10. Higgins, E. P. C.; Papaderakis, A. A.; Byrne, C.; Walton, A. S.; Lewis, D. J.; Dryfe, R. A. W., Intrinsic effects of thickness, surface chemistry and electroactive area on nanostructured MoS2 electrodes with superior stability for hydrogen evolution. *Electrochimica Acta* **2021,** *382*, 138257.

11. Castro, S. L.; Bailey, S. G.; Raffaelle, R. P.; Banger, K. K.; Hepp, A. F., Nanocrystalline Chalcopyrite Materials (CuInS2 and CuInSe2) via Low-Temperature Pyrolysis of Molecular Single-Source Precursors. *Chemistry of Materials* **2003,** *15* (16), 3142-3147.

12. Shen, S.; Zhang, Y.; Peng, L.; Xu, B.; Du, Y.; Deng, M.; Xu, H.; Wang, Q., Generalized synthesis of metal sulfide nanocrystals from single-source precursors: size, shape and chemical composition control and their properties. *CrystEngComm* **2011,** *13* (14), 4572-4579.

13. Liu, R.; Chen, H.; Zhao, K.; Qin, Y.; Jiang, B.; Zhang, T.; Sha, G.; Shi, X.; Uher, C.; Zhang, W.; Chen, L., Entropy as a Gene-Like Performance Indicator Promoting Thermoelectric Materials. *Adv Mater* **2017,** *29* (38).

14. <http://abulafia.mt.ic.ac.uk/shannon/ptable.php>.

15. Zeng, N.; Hopkinson, D. G.; Spencer, B. F.; McAdams, S. G.; Tedstone, A. A.; Haigh, S. J.; Lewis, D. J., Direct synthesis of MoS2 or MoO3via thermolysis of a dialkyl dithiocarbamato molybdenum(iv) complex. *Chemical Communications* **2019,** *55* (1), 99-102.

16. Rong, J.; Xu, J.; Qiu, F.; Fang, Y.; Zhang, T.; Zhu, Y., 2D metal-organic frameworks-derived preparation of layered CuS@C as an efficient and stable electrocatalyst for hydrogen evolution reaction. *Electrochimica Acta* **2019,** *323*, 134856.

17. Ren, H.; Xu, W.; Zhu, S.; Cui, Z.; Yang, X.; Inoue, A., Synthesis and properties of nanoporous Ag2S/CuS catalyst for hydrogen evolution reaction. *Electrochimica Acta* **2016,** *190*, 221-228.

18. Basu, M.; Nazir, R.; Mahala, C.; Fageria, P.; Chaudhary, S.; Gangopadhyay, S.; Pande, S., Ag2S/Ag Heterostructure: A Promising Electrocatalyst for the Hydrogen Evolution Reaction. *Langmuir* **2017,** *33* (13), 3178-3186.

19. Wei, Y.; He, W.; Sun, P.; Yin, J.; Deng, X.; Xu, X., Synthesis of hollow Cu/Cu2O/Cu2S nanotubes for enhanced electrocatalytic hydrogen evolution. *Applied Surface Science* **2019,** *476*, 966-971.

20. Pataniya, P. M.; Patel, V.; Sahatiya, P.; Late, D. J.; Sumesh, C. K., Hydrogen evolution reaction in acidic and basic medium on robust cobalt sulphide electrocatalyst. *Surfaces and Interfaces* **2022,** *34*, 102319.

21. Aqueel Ahmed, A. T.; Sekar, S.; Lee, S.; Im, H.; Preethi, V.; Ansari, A. S., Nitrogen-doped cobalt sulfide as an efficient electrocatalyst for hydrogen evolution reaction in alkaline and acidic media. *International Journal of Hydrogen Energy* **2022,** *47* (95), 40340-40348.

22. Ge, Y.; Wu, J.; Xu, X.; Ye, M.; Shen, J., Facile synthesis of CoNi2S4 and CuCo2S4 with different morphologies as prominent catalysts for hydrogen evolution reaction. *International Journal of Hydrogen Energy* **2016,** *41* (44), 19847-19854.

1. Cyclic voltammograms were recorded on glassy carbon electrodes with 2.5 mM of each precursor in acetone using 0.1 M [TBA][PF_6_] supporting electrolyte. [↑](#footnote-ref-2)
